# The costs and the potential allocation of costs of bouillon fortification: The cases of Nigeria, Senegal, and Burkina Faso

**DOI:** 10.1111/nyas.15234

**Published:** 2024-10-21

**Authors:** Stephen A. Vosti, Michael Jarvis, Olufolakemi Mercy Anjorin, Reina Engle‐Stone, Maguette Beye, Faith Ishaya, Karim Koudougou, Blessing Oni, Hervé Somda, Katherine P. Adams

**Affiliations:** ^1^ Department of Agricultural and Resource Economics University of California Davis Davis California USA; ^2^ Institute for Global Nutrition, Department of Nutrition University of California Davis Davis California USA; ^3^ Consultant Lagos Nigeria; ^4^ Helen Keller International Dakar Senegal; ^5^ Helen Keller International Abuja Nigeria; ^6^ Helen Keller International Ouagadougou Burkina Faso; ^7^ Consultant Ouagadougou Burkina Faso

**Keywords:** bouillon fortification, Burkina Faso, cost allocations, costs, large‐scale food fortification, Nigeria, Senegal

## Abstract

Food and condiment fortification programs are needed to address micronutrient deficiencies and their health, developmental, and mortality consequences; but these programs are never free. Knowing program costs and their allocation across stakeholders is essential to design and manage effective, efficient, and sustainable programs. We developed 10‐year hypothetical bouillon fortification program cost models for Nigeria, Senegal, and Burkina Faso that included start‐up and operational costs for government and industry, as well as premix costs generated by an embedded premix cost calculator to allow for alternative premix formulas in cost calculations. The main drivers of total costs were total bouillon consumption and the types and amounts of fortificants in the micronutrient premix. For a premix that meets 30% of Codex Nutrient Reference Values in 2.5 g of bouillon for vitamin A, folate, vitamin B12, zinc, and iron, the cost per metric ton of fortified bouillon was ∼$325 for all countries (∼$0.01 per 2.5 g serving). Annual start‐up costs ranged from ∼$324k (Burkina Faso) to ∼$455k (Nigeria); nonpremix annual operating costs ranged from ∼$108k (Burkina Faso) to ∼$3.9m (Nigeria); and annual premix costs varied from ∼$2.4m (Burkina Faso) to ∼$76m (Nigeria). In policy discussions, program costs should be set alongside nutritional benefits.

## Graphical abstract

Bouillon fortification program costs can be substantial and depend largely on national bouillon consumption and the micronutrient content of premixes. The MINIMOD cost model can estimate program start‐up costs and annual fortification program costs, including premix costs. Stakeholders must agree on how all program costs will be paid.

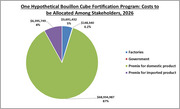

## INTRODUCTION

Micronutrient deficiencies are common in low‐ and middle‐income countries (LMICs) with consequences for health, growth, and child development, and can influence childhood mortality via impacts on the prevalence of neural tube defects (relevant for folate), mortality from diarrhea (vitamin A and zinc), and pneumonia (zinc).^1^, ^2^, ^3^, ^4^, ^5^ One important pathway for addressing micronutrient deficiencies is by enriching diets with micronutrients. But micronutrient deficiencies are not the only nutrition problems faced by these populations. Stunting and wasting are also important problems^5^ along with noncommunicable diseases such as hypertension,^6^ with potential but incomplete overlaps with micronutrient deficiencies. There are other problems in the domains of public health (e.g., neglected tropical diseases^7^) that may also merit attention. Therefore, even within the fairly narrow domain of public health and nutrition, there are many pressing problems; addressing each of these will require financial and human resources and there are never sufficient resources to address all problems, so hard choices have to be made regarding which problems to address and at what levels, as well as which to tackle when/if additional resources become available.

In this complex nutrition decision‐making process that also involves issues related to equity, technical feasibility, and politics, a pair of key factors is (a) the costs associated with alternative investments available to address specific problems, and (b) which stakeholders will cover which portions of these costs. One underlying premise of this paper is that all intervention program costs must (eventually) be paid by one or more stakeholder groups. Failure to do so will generate relatively ineffective and unsustainable intervention programs. With information on these key factors, decision‐makers will be in a better position to set the expected nutritional and other benefits of addressing micronutrient deficiencies alongside the costs of alternative intervention programs, and thereby include evidence on efficiency and affordability in their policy discussions.

Large‐scale food fortification (LSFF) programs have been identified as potentially cost‐effective strategies for addressing some micronutrient deficiencies.^8^ Indeed, all of the countries included in this study—Burkina Faso, Nigeria, and Senegal—have ongoing LSFF programs, for example, Nigeria has five programs delivering vitamin A (wheat flour, maize flour, refined edible oils, margarine, and sugar). While these programs all contribute to reducing the prevalence of micronutrient inadequacies, modeling in these country contexts of vitamin A, iron, zinc, folate, and vitamin B12 suggests that the combined effects of all LSFF programs performing at current levels of compliance with published standards are generally not sufficient to eliminate the public health problem.^9^, ^10^, ^11^ Therefore, additional delivery vehicles are needed, and given consumption patterns,^12^, ^13^ bouillon is an excellent candidate for many countries in West Africa. The objectives of this paper were to estimate the costs associated with alternative hypothetical large‐scale bouillon fortification programs in Burkina Faso, Nigeria, and Senegal, with special attention paid to specific cost categories (e.g., start‐up vs. operational costs) and the stakeholder groups that might be called upon to pay them.

Other papers provide evidence on the expected nutritional and child mortality–reducing benefits associated with these programs,^9^, ^10^, ^11^, ^14^ and another paper links these benefits and costs to provide evidence on cost‐effectiveness.^15^

The next section introduces the Micronutrient Intervention Modeling (MINIMOD) LSFF cost model methodology and the data that support it. We also report the estimated costs of 10‐year, multi‐fortified bouillon fortification programs for Senegal, Burkina Faso, and Nigeria, with special attention paid to stakeholder‐specific costs during the 2‐year start‐up and 8‐year operational periods, as well as to premix costs—the largest cost share in most LSFF programs.^16^, ^17^ A broad array of national and international stakeholders has historically contributed to covering some of the costs of LSFF programs, especially during the start‐up phase.^16^

## METHODS AND DATA

### Methods

**FIGURE 1:**
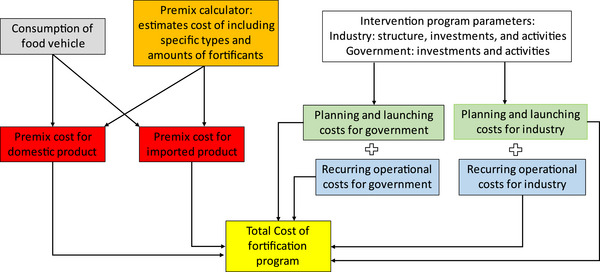
Structure of the MINIMOD large‐scale food fortification program cost model.

An ingredient‐ and activity‐based approach was used to estimate the economic costs (out‐of‐pocket costs plus opportunity costs, e.g., the value of time dedicated to specific activities) of planning, designing, launching, and operating bouillon fortification programs in Burkina Faso,^a^ Senegal, and Nigeria from a societal perspective. A social perspective includes the out‐of‐pocket and opportunity costs of all stakeholders in society. It is very important to note, from the outset, that the approach described below focuses exclusively on the marginal costs of fortification for governments and for industry, that is, we do not address the costs of other ingredients (e.g., salt and flavoring) and activities (e.g., overall factory management) required to produce bouillon cubes. Figure 1 provides an overview of this approach undertaken once the micronutrient‐specific assessments of deficiency and/or gaps in nutrient adequacy have been completed.

The first step in generating estimates of the costs of any bouillon fortification program is to estimate the average annual consumption of bouillon cubes (gray box). Next, decisions need to be made regarding which micronutrients, and the amounts of each, to include in the premix (orange box)—these decisions can have large implications for overall program costs. Not all countries produce all of the bouillon that they consume; indeed, all countries import some bouillon products and some countries (e.g., Burkina Faso) import all of the bouillon they consume. Since monitoring and regulatory activities and their associated costs are different for nationally produced versus imported products, the cost model splits the total supply of bouillon between national production and imported products (red boxes). It is important to note that domestic and international producers of bouillon are expected to comply with national standards; in the model, both sets of suppliers are assumed to adhere to these standards at the same levels of compliance, and for any given country, the level of compliance does not vary over time. The size and age structure of the national population, and hence the consumer base for bouillon products, are the only model parameters that vary over time. All other technical parameters and unit values (e.g., fortificant prices and wage rates) are held constant throughout the 10‐year model time horizon. The model could be easily adapted to incorporate temporal variation in these key parameters and unit values, but credible estimates of them in the future would be required.

Programmatic, nonpremix costs are faced by governments and by industries (white box), both in the start‐up phase (during which no fortification occurs and hence no nutritional or mortality benefits accrue [green boxes]) and in the operational phase of the fortification program (blue boxes). The total cost of the bouillon fortification program is the sum of all of these costs (yellow box). All of these costs are assessed on an annual time‐step and summed over the temporal extent of the model, which is 10 years.^b^ A 10‐year planning time horizon is adopted to ensure that all hypothetical programs, including those envisioning improvements to existing programs, have sufficient time to deliver micronutrients and generate the associated nutritional benefits, and no discount rate is applied.

The boxes in Figure 1 give rise to a system of equations associated with start‐up and operational activities for firms and governments—and for premix costs—that sum to the total program costs each year and over the decision time horizon. From here on, we refer to planning and launching costs as “start‐up costs” and to recurring operational costs as “operational costs.”

More specifically, Equation (1) focuses on start‐up costs for firms (*SUC^F^*). It identifies the *j^th^* start‐up investment (there are *J* of these) such as new equipment (*sufinv*) and their unit costs (*Psufinv*) and the *i^th^* start‐up activities^c^ (there are *N* of these) such as training and modifications to product labels (*sufa*) that a given firm *f* would undertake and the activity‐specific prices (*Psufa*) that firms would face for each activity in a given year *t* during the start‐up period, which is assumed to run for 2 years. Activities and their prices are multiplied to arrive at activity‐specific costs, which are then summed across all activities and over time. These costs are then summed over the *f* firms in the industry, which is comprised of *F* firms, all of which are assumed to face the same activity‐specific unit costs.

(1) $$\mathrm{SU}C^{F}=\sum_{f=1}^{f=F}\;\;\sum_{t\;=\;1}^{t\;=\;2}\;\sum_{j\;=\;1}^{j\;=\;J}\;\sum_{i\;=\;1}^{i\;=\;N}\;\left(\mathrm{sufin}v_{f,j,t}*\mathrm{Psuf}\mathrm{in}v_{j,t}+\mathrm{suf}a_{f,i,t}*\mathrm{Psuf}a_{i,t}\;\right)\;$$

Symmetrically, Equation (2) focuses on start‐up costs for government (*SUC^G^*). It identifies the *j* start‐up investments such as new equipment (*suginv*) and their unit costs (*Psuginv*) and *i* start‐up activities (*suga*) that governments^d^ or their representatives would undertake and the activity‐specific prices (*Psuga*)^e^ that governments would face in a given year *t* during the start‐up period, which again runs for 2 years. Activities—which, as above, contain all of the ingredients and their respective unit costs—and their prices are multiplied to arrive at activity‐specific costs, which are then summed across all activities and over time.

(2) $$\mathrm{SU}C^{G}=\sum_{t=1}^{t=2}\;\sum_{j=1}^{j=J}\;\;\sum_{i\;=\;1}^{i\;=\;N}\left(\mathrm{sugin}v_{j,\;t}*\mathrm{Psug}\mathrm{in}v_{j,t}+\;\mathrm{sug}a_{i,t}*\mathrm{Psug}a_{i,t}\;\right)\;$$

Once the start‐up period is completed, operational costs begin for both firms (*OPC^F^*) and government (*OPC^G^*). Equation (3) identifies the *m^th^* operational activities (*opfa*) (there are *M* of these) that a given firm *f* would undertake and the activity‐specific prices (*Popfa*) that firms would face for each activity in a given year *t* during the operational period. Activities and their prices are multiplied to arrive at activity‐specific costs, which are then summed across all activities and over time. These costs are then summed over the *f* firms in the industry, which is comprised of *F* firms.

(3) $$\mathrm{OP}C^{F}=\sum_{f=1}^{f=F}\;\;\sum_{t\;=\;3}^{t\;=\;10}\;\sum_{m\;=\;1}^{m\;=\;M}\left(\mathrm{opf}a_{f,m,t}*\mathrm{Popf}a_{m,t}\;\right)\;$$

Equation (4) does the same for governments, identifying the *m* operational activities (*opga*) that governments would undertake and the activity‐specific prices (*Popga*) that governments would face for each activity in a given year *t* during the operational period. Activities and their prices are multiplied to arrive at activity‐specific costs, which are then summed across all activities and over time.

(4) $$\mathrm{OP}C^{G}=\sum_{t=3}^{t=10}\;\;\sum_{m\;=\;1}^{m\;=\;M}\left(\mathrm{opg}a_{m,t}*\mathrm{Popg}a_{m,t}\;\right)\;$$

The total quantities of the various micronutrients (*mn*_*k,t*_) that will be imported in Equation (5) will depend on the size of the consuming population (*CONSUMPOP*), average amounts of bouillon consumption (*AVGCON*), and adherence by firms to the standards that set the amounts of each micronutrient that should be included in each serving *(ADHSTND*). Note that only the size of the consuming population changes over time; average consumption amounts per consumer and adherence to the fortification levels contained in the standard are assumed to remain constant over the entire simulation period. In what follows, we have fixed the premix formula for each country at 30% of Codex nutrient reference values (NRVs) for adults consuming 2.5 g/day, so the free variables in the estimates of the cost of premix per kilogram are fortificant prices. Thirty percent NRVs was chosen because it is consistent with Codex guidelines for “High in” nutrient content claims. Other levels of fortification were modeled for analysis in several other papers;^9^, ^10^, ^11^, ^14^ the cost models for each country can be used to generate estimates of all of these alternative premix formulas.

(5) $$mn_{k,t}=CONSUMPOP_{t}*AVGCON*ADHSTND_{k}$$

Once the amounts of given micronutrients (*mn_k_*) have been identified, Equation (6) calculates premix cost (*PMX*) by multiplying *mn_k_* by their micronutrient‐specific prices (*Ppm_k_*) and summed over all micronutrients in the premix and over time to arrive at total premix cost.

(6) $$PMX\;=\sum_{t=3}^{T=10}\;\;\sum_{k\;=\;1}^{k\;=\;K}\left(mn_{k,t}*Ppm_{k,t}\right)\;$$

Finally, for Equation (7), the five core ingredients to bouillon fortification program costs—start‐up costs for firms and for governments (*SUC^F^* and *SUC^G^*), operational costs for firms and governments (*OPC^F^* and *OPC^G^*), and premix costs (*PMX*)—are summed to arrive at total program costs (TPC) over the 10‐year planning time horizon. The total amount and costs of each micronutrient consumed by the national population are included in this calculation, regardless of whether the product is produced nationally or imported.

(7) $$\mathrm{TPC}=SUC^{F}+SUC^{G}+OPC^{F}+OPC^{G}+PMX$$

### Data

The fortified bouillon cost models developed for Nigeria, Burkina Faso, and Senegal are essentially hypothetical, but they build on existing programs and production processes that are well understood. For example, while no country currently has a mandatory bouillon fortification program in place, many countries have operating LSFF programs focused on other staple foods or condiments (core information on these programs is available at https://fortificationdata.org/), and the institutions and mechanisms to monitor and evaluate nationally produced and imported fortified products. Governments envision weaving fortified bouillon programs into these existing programs and systems,^19^ therefore, existing programs provide a basis for government cost model development. Also, some firms have begun to fortify bouillon cubes with single micronutrients (in addition to iodine), so industrial bouillon fortification processes, and the internal quality assurance and quality control activities imbedded in them, are also known and can provide a point of departure for cost model development models for firms. A multi‐fortified bouillon cube containing vitamin A, vitamins B9 and B12, iron, and zinc was produced and tested for acceptability in Ghana,^21^ and was used in a randomized, controlled trial in Tamale, Ghana.

All cost models were codeveloped in association with in‐country teams drawn from government, industry, independent consultants, academia, and nongovernmental organization. These teams reviewed lists of placeholder model parameters and worked with the MINIMOD team to update and upgrade them. They reviewed and provided guidance on all of the underlying assumptions that were built into the cost models and helped identify relevant sensitivity analyses.

Cost model development benefited from two factors. First, the MINIMOD team has produced suites of LSFF program cost models for other countries (also see the MINIMOD project website for details https://minimod.ucdavis.edu/).^20^ These models have the same basic structure (see Figure 1) as those developed for the countries examined here, although the details vary by fortifiable product and by country. Second, the resurgence of interest in using bouillon cubes as a delivery vehicle for micronutrients has generated a series of recent evaluations of industry and government preparedness and needs associated with bouillon fortification,^18^, ^19^ and these assessments also provided information on cost model parameters.

Finally, an Excel‐based tool was developed to estimate the unit price of different micronutrient premix formulations. Populated with data on the unit prices of fortificants, addition rates, overages required to compensate for fortificant losses, industry mark‐ups, and so on, a user can then estimate the per kilogram costs of alternative micronutrient formulations. Table 1, borrowing the structure set out in Figure 1, identifies the sources of data and the literature and key informants that were tapped to populate the many model parameters required to develop the cost models for each country.

**TABLE 1:** Data sources for bouillon cube fortification cost model.

Cost model components	Data sources	Notes
Consumption of food vehicle		
Population size	United Nations Population Project, 2019 Update	
% consuming bouillon	HCES^a^ data 2018−2019	As reported in Adams et al.^9^, ^10^, ^11^
Average amount of bouillon consumed	HCES^a^ data 2018−2019	As reported in Adams et al.^9^, ^10^, ^11^
Structure of industry		
Number of firms	Nigeria: Information on industry structure in Nigeria was derived from key informant interviews undertaken over the period 2021−2022. Wage rates for personnel were set at 250% of the average wage rates in Nigeria in 2021; for factory laborers, the base rate was $4038/year, and for technical personnel, the base rate was $5047/year). Average wage is calculated by multiplying GDP per working person^22^ by individual labor share of GDP.^23^ Personal communication with in‐country consultants (2021)	Bouillon cubes are not currently produced in Burkina Faso; some national product occurs in Senegal. Country‐specific cost models account for the % of national/imported cubes, and the firm and government M&E costs associated with each source of bouillon.
% of bouillon nationally produced	Nigeria: Personal communication with in‐country consultant (2021)	Burkina Faso and Senegal 10% assumed to reflect the start of fortification within the country, for the purpose of the analysis.
Premix cost calculator		
% of micronutrient in fortificants	Personal communication with industry expert (2023)	
Addition rates	Personal communication with industry expert (2023)	
Fortificant prices	Personal communication with industry expert (2023)	
Planning and launching costs: Government		
Nonlabor input parameters	Fiedler and MacDonald^24^ Fiedler and Afidra^25^	Supplemented with assumptions provided by in‐country collaborators.
Nonlabor unit prices	UNICEF Supply Catalog^26^ and BioAnalyt website^27^	For prices of equipment
Labor input parameters	Assumptions	For number of training sessions
Labor unit prices	Fiedler and Afidra^25^	For cost of training sessions
Planning and launching costs: Industry		
Nonlabor input parameters	Fiedler and MacDonald^24^ Fiedler and Afidra^25^	Supplemented with assumptions provided by in‐country collaborators
Nonlabor unit prices	UNICEF Supply Catalog^26^	For prices of equipment
Labor input parameters	Assumptions	For number of training sessions
Labor unit prices	Fiedler and Afidra^25^	For cost of training sessions
Operational costs: Government		
Nonlabor input parameters	Fiedler and Macdonald^24^ Personal communication with industry expert (2023)	Supplemented with assumptions provided by in‐country collaborators
Nonlabor unit prices	Fiedler and Macdonald^24^ UNICEF Supply Catalog^26^ and BioAnalyt website^27^	Supply catalog for testing supply prices
Labor input parameters	Personal communication with industry expert (2023)	
Labor unit prices	Assumptions using calculated average annual wage (per working person)	Confirmed to be accurate with in‐country collaborators
Operational costs: Industry		
Nonlabor input parameters	Fiedler and Macdonald^24^ Personal communication with industry expert (2023)	Supplemented with assumptions provided by in‐country collaborators
Nonlabor unit prices	Fiedler and Macdonald^24^ UNICEF Supply Catalog^26^ and BioAnalyt website^27^	Supply catalog for testing supply prices
Labor input parameters	Personal communication with industry expert (2023)	
Labor unit prices	Assumptions using calculated average annual wage (per working person)	Confirmed to be accurate with in‐country collaborators

^a^ Household Consumption and Expenditure Surveys.^28^, ^29^, ^30^

## RESULTS

### Bouillon consumption

Total bouillon consumption in any setting depends on the size and bouillon‐consumption habits of the population. Table 2 reports the sizes of the Nigerian, Burkinabe, and Senegalese populations, nationally and by geopolitical zones, for 2021. Roughly 211 million people inhabit Nigeria.^31^ Bouillon consumption is widespread and spatially very uniform in that country; 98% of the national population reported consuming bouillon in the 7 days prior to the 2021 National Food Consumption and Micronutrient Survey, ranging from 100% in the South South zone to 96% in the North East.^32^ National average consumption among consumers was 3.6 g/day per woman of reproductive age (WRA), and ranged from 4.6 g/day in the North Central zone to 2.6 g/day in the South West zone.^f^ This led to ∼768 Mg of bouillon consumed per day in 2021, all of which, in theory, would have to be fortified under a mandatory fortification program. In practice, we assume that 75% of bouillon cubes are fortified to 100% of the standard (i.e., 75% of all bouillon products, regardless of location of production, are fortified to 100% of the standard, and 25% are not fortified at all). This assumption applies to nationally produced and imported bouillon cubes.

**TABLE 2:** Population size and bouillon consumption in Nigeria, Burkina Faso, and Senegal, by geographic region.

	Population (2021)	Subnational share of total population (proportion)	Bouillon cube reach (% reporting consumption)	Bouillon cube consumption (g/day/WRA among consumers)	Total bouillon cube consumption (g/day)
**Nigeria** ^a^	**211,400,704**		**98%**	**3.65**	**768,064,140**
North Central	31,958,461	0.1512	100%	4.62	147,048,548
North East	28,693,458	0.1357	96%	3.73	103,217,865
North West	53,469,815	0.2529	98%	3.91	206,027,976
South East	23,986,444	0.1135	96%	3.86	89,320,422
South South	31,496,200	0.1490	100%	3.71	116,654,272
South West	41,796,326	0.1977	98%	2.58	105,795,057
					
**Burkina Faso** ^b^	**21,497,097**		**82%**	**1.45**	**25,227,611**
Boucle de Mouhoun	2,085,064	0.0970	81%	1.80	3,052,052
Cascades	880,150	0.0409	70%	1.84	1,136,293
Centre	3,078,501	0.1432	74%	1.38	3,118,157
Centre‐Est	1,703,773	0.0793	81%	1.88	2,592,276
Centre‐Nord	1,785,995	0.0831	89%	1.10	1,756,964
Centre‐Ouest	1,736,140	0.0808	89%	0.90	1,385,368
Centre‐Sud	919,122	0.0428	85%	1.81	1,420,858
Est	1,890,662	0.0879	80%	1.34	2,025,642
Hauts Bassins	2,296,099	0.1068	80%	1.82	3,359,004
Nord	1,723,016	0.0802	80%	0.71	982,342
Plateau Central	1,001,496	0.0466	87%	1.27	1,105,798
Sahel	1,480,642	0.0689	95%	1.87	2,630,189
Sud‐Ouest	916,436	0.0426	84%	0.86	662,668
					
**Senegal** ^c^	**17,196,308**	**0.2322**	**90%**	**2.30**	**34,442,025**
Dakar	3,993,584	0.0407	84%	2.45	8,184,747
Ziguinchor	699,058	0.1109	94%	2.32	1,519,580
Diourbel	1,906,228	0.0673	91%	2.79	4,825,548
Saint‐Louis	1,157,064	0.0504	94%	2.29	2,481,375
Tambacounda	867,293	0.0711	90%	2.20	1,711,426
Kaolack	1,223,174	0.1324	94%	1.62	1,863,717
Thiès	2,277,186	0.0647	88%	2.46	4,901,369
Louga	1,112,829	0.0529	92%	2.34	2,402,452
Fatick	909,406	0.0490	94%	2.21	1,890,577
Kolda	843,291	0.0416	96%	1.75	1,422,783
Matam	716,100	0.0420	93%	1.87	1,246,321
Kaffrine	721,769	0.0112	89%	1.49	951,576
Kédougou	192,674	0.0335	92%	5.86	1,040,552
Sédhiou	576,652	0.2322	94%	1.60	871,477

Abbreviation: WRA, woman of reproductive age.

^a^ 2021 National Food Consumption and Micronutrient Survey Preliminary Report and household‐level bouillon consumption data collected on behalf of the Nigeria Bouillon Country Working Group in 2020; Adams et al. 2024.^11^

^b^ Burkina Faso 2018/2019 Enquête Harmonisée sur les Conditions de Vie des Ménages^28^; Adams et al.^10^

^c^ Senegal 2018/2019 Enquête Harmonisée sur les Conditions de Vie des Ménages^29^; Adams et al.^9^

Table 2 also reports the same evidence for Burkina Faso and Senegal, respectively. Several factors merit mention when comparing across countries. First, the population sizes differ markedly (Senegal has less than 10% of the Nigerian population). Second, the reach of bouillon also differs across countries; Nigeria registers the highest and most spatially uniform pattern of bouillon consumption, followed by Senegal, and lastly Burkina Faso. Reach and average consumption of bouillon will combine to influence the expected benefits (nationally and subnationally) of bouillon fortification programs, as well as their cost.

### Industry structure and characteristics, and government regulatory activities

The industry structure and characteristics of a given delivery vehicle will influence the costs faced by industry and by government. Table 3 reports the relevant industry characteristics for bouillon in Nigeria. Note the relatively small number of large‐scale bouillon production facilities, the proposed (by regulatory mandate) number of factory and port‐of‐entry inspections, and the very large proportion of bouillon cubes that are produced nationally.

**TABLE 3:** Structure and characteristics of the bouillon industry in Nigeria.

Bouillon cube industry information	
Number of bouillon cube factories	8
Average number of days of bouillon cube production per factory per year	250
Average wage of factory labor employee ($/year)	$10,094
Average wage of factory lab or quality assurance employee ($/year)	$12,617

Government monitoring and evaluation (M&E) information	
Number of industrial‐scale bouillon cube factories	8
Number of on‐site factory inspections (per factory per year)	12
Number of imported bouillon cube ports of entry	10
Number of imported bouillon cube consignments/deliveries to be inspected (per port of entry per year)	25
Number of commercial site visits for commercial monitoring (total number per monitoring year)	300
Average wage of import monitor ($/year)	$12,617
Average wage of commercial monitor ($/year)	$12,617

Structure of consumption	
% of total national consumption that is produced domestically	90%
% of total domestic production that is fortifiable/industrially produced	100%
% of total national consumption that is imported	10%
% of total imports that is fortifiable/industrially produced	100%

*Note*: Values are from the authors’ calculations. All monetary values are expressed in 2021 USD.

It is important to note that the structure of the bouillon industries in Burkina Faso and Senegal are markedly different from that of Nigeria (see Tables S1 and S2). Ninety percent of all bouillon consumed in Senegal and Burkina Faso is assumed to be imported, with the remaining 10% being produced nationally; the implications of this assumption for total cost are small.^g^ Therefore, in both countries, monitoring and evaluation of imported products will be a larger share of overall government expenditures than is the case in Nigeria, and a much smaller share of total premix supply management costs will be paid by national firms. That said, all premix costs will have to be paid, and consumers are assumed to cover these costs via increases in retail prices.

### Premix costs

Micronutrient premixes represent the major cost of bouillon fortification, but can vary considerably depending on the content of premix (i.e., the number, types, and amounts of fortificants included in the premix). Table 4 identifies the information required to estimate premix costs. In this example, we focus on a single premix calculated to meet 30% of Codex Nutrient Reference Values (NRVs) for iron, vitamin A, zinc, vitamin B12, and folate, for adults consuming 2.5 g/day of bouillon.^h^ The first and second columns of Table 4 identify the nutrients and their specific fortificants (i.e., the chemical form that serves as the micronutrient source). The activity level column reports the proportion (by weight) of each micronutrient in each fortificant. The target fortification levels appear in the fourth column, followed by the overage estimated to be required to compensate for losses in vitamins between the time of fortification and the time that the fortified product is consumed. Normally, the standards for fortified foods and condiments include overages in calculating target levels of fortification. Since no standards currently exist for bouillon cubes, we include the targeted amounts of each micronutrient to be consumed and the overage required to address expected fortificant degradation.

**TABLE 4:** MINIMOD Premix Cost Calculator results for fortification of bouillon with five micronutrients at concentrations that provide 30% of Codex Nutrient Reference Values in 2.5 g bouillon.

Nutrient	Compound	Activity (%)	Fortification level (mg/kg cube)	Overage (mg/kg)	Amount fortificant (mg/kg cube)	Proportion of fortificant in premix	Price ($/kg)	Cost ($/kg premix)
Iron	Micronized ferric pyrophosphate	25.0%	2640		10,560.0	0.65	11.50	$7.47
Vitamin A	Retinyl palmitate: 250,000 IU/g (dry)	7.5%	96	29	1664.0	0.10	55.00	$5.63
Zinc	Zinc oxide	80.0%	1680		2100.0	0.13	7.00	$0.90
B12	Vitamin B‐12 0.1% WS	0.1%	0.288	0.086	374.4	0.02	45.00	$1.04
Folic acid	Folic acid	90.0%	28.8	8.6	41.6	0.003	65.00	$0.17
Nutrients subtotal					14,740.0	0.91		$15.21
110% of subtotal					16,214.0			
Excipient (filler)					1510.0	0.09	1.50	$0.14
Total					16,250	1.00		
			Addition rate		16,250			
								
Nutrients total ($/kg)								$15.35
Up‐charge ($/kg)								$1.00
Total ($/kg cube)								$0.27
**Total cost per kg of premix**								**$16.35**
**Total cost per MT of fortified cube**								**$265.73**

*Note*: Values are for Nigeria, Burkina Faso, and Senegal, and were calculated by the authors. All monetary values are expressed in 2021 USD.

Abbreviation: MT, metric ton.

The amount of each fortificant to be included in the cube (considering the target level of fortification, the overage, and the activity level) is then multiplied by the price^i^ of the fortificant to arrive at the cost of each fortificant in each kg of premix. For this premix formula, the total cost of premix per metric ton (MT) of bouillon is ∼$266. Note that the contributions to total cost vary substantially across micronutrients. For example, in these estimates, iron accounted for almost half the total cost of the five fortificants in the premix, and vitamin A accounted for more than one‐third. Note also that the cost per MT of this specific bouillon will not vary across countries or over time. However, the total cost of producing the fortified product may vary depending on country‐specific production and the monitoring and evaluation costs. The total cost of the program will certainly vary based on population size, bouillon consumption habits, and the structure of the national bouillon industry. Fortificant prices are assumed to remain constant over the simulation timeframe. We undertook sensitivity analyses to assess the impacts of this fixed‐price assumption on program costs (see Table S14).

Table 5 uses the results of the premix calculator plus other model parameters to estimate the annual cost of premix for domestically produced fortified bouillon cubes (again, using 30% of Codex NRVs, as an example) in the context of Nigeria, including import duties.^j^ Note that premix costs are only encountered during the operational phase of the fortification program (2023–2030).

**TABLE 5:** Summary of premix^a^ costs for domestic production of multiple micronutrient‐fortified bouillon from 2023 to 2030 for Nigeria, 30% of Codex NRVs.

	2023	2024	2025	2026	2027	2028	2029	2030
Availability of bouillon cubes in food supply (g/capita/day)	3.63	3.63	3.63	3.63	3.63	3.63	3.63	3.63
Total population (000s)	222,182	227,713	233,343	239,073	244,902	250,830	256,855	262,977
Annual volume of food vehicle (MT)	294,641	301,976	309,442	317,041	324,771	332,631	340,622	348,740
% national consumption produced domestically	90%	90%	90%	90%	90%	90%	90%	90%
% total domestic production that is industrially produced/fortifiable	100%	100%	100%	100%	100%	100%	100%	100%
% fortifiable domestic product that is fortified	75%	75%	75%	75%	75%	75%	75%	75%
Annual volume of fortified food vehicle (MT)	198,883	203,834	208,873	214,002	219,220	224,526	229,920	235,400
Transportation/distribution/storage ($/kg)	0.20	0.20	0.20	0.20	0.20	0.20	0.20	0.20
Tax or duty on imported premix (%)	20%	20%	20%	20%	20%	20%	20%	20%
Cost per kg of premix	$19.82	$19.82	$19.82	$19.82	$19.82	$19.82	$19.82	$19.82
Premix cost per MT of cubes, including shipping, taxes, etc.	$322.12	$322.12	$322.12	$322.12	$322.12	$322.12	$322.12	$322.12
**Total premix cost for domestic food vehicle (000s)**	$64,065	$65,659	$67,283	$68,935	$70,616	$72,325	$74,062	$75,828

*Note*: Values are from the authors’ calculations. All monetary values are expressed in 2021 USD.

Abbreviation: MT, metric tons.

^a^ Assuming 30% of Codex NRV of vitamin A, B9, B12, zinc, and iron, in 2.5 g of bouillon.

Companion submatrices in the MINIMOD cost model exist for the premix costs for imported bouillon products (see the Supplementary Material). All imported products are assumed to adhere to the same fortification standards that national producers must adhere to (i.e., 75% of products adhere to 100% of the standards), and we assume that these fortification costs will be passed onto bouillon consumers, hence the inclusion of premix costs of imported products in the overall national estimates of fortification costs.^k^ Due to porous borders and imperfect regulatory processes, we do not expect that all of the bouillon products in any country at any given point in time will be fortified to the assumed standards. The failure to reach 100% compliance has cost consequences for firms; lower amounts and values of premixes need to be purchased, managed, and so on, with knock‐on consequences for the costs that need to be passed on to consumers. Government costs, on the other hand, remain the same, regardless of adherence to standards; improving adherence to standards would, however, require additional government investments and increased operational expenses.

For Nigeria, the annual average cost of premix required for this hypothetical fortification program (domestic production plus imports) operating at 75% compliance is ∼$76 million USD.

### Industry and government start‐up costs: Cost calculation ingredients

Planning, investments, and training are required to prepare for the production of fortified bouillon cubes. Key factory investments include feeders to blend premix and testing equipment to undertake product quality testing; all equipment is assumed to be imported and hence subject to import taxes. In addition, new bouillon formulations need to be designed and produced, product labels need to be updated, and employees need to be trained in new quality assurance practices. Most investments are assumed to take place during the first of 2 start‐up years, with some training activities undertaken in the second year of the start‐up period (Table 6, top half).

**TABLE 6:** Start‐up bouillon fortification program costs for factories and the government in Nigeria.

Bouillon cube start‐up costs: Factory	2021	2022
*Equipment*		
Feeder (unit cost) and installation	$5000	
Number of factories requiring a feeder	8	
Total feeder cost	$40,000	
iCheck portable fluorometer	$8690	
iCheck portable photometer	$3373	
Manual centrifuge	$476	
Number of factories requiring a fluorometer	8	
Number of factories requiring a photometer	8	
Number of factories requiring a centrifuge	8	
Total fluorometer, photometer, and centrifuge costs	$100,312	
Tax or duty on factory equipment (%)	10%	
Equipment shipping (%)	10%	
VAT (%)	8%	
**Total equipment cost**	$179,599	
*Labeling*		
Redesign of labels and label printing plates (unit cost)	$327	
Number of factories/brands requiring label and printing plates redesign	8	
**Total labeling cost**	$2617	
*Training*		
Training and sensitization for fortification and quality assurance personnel (unit cost per training session)	$249	$249
Number of training sessions per factory	3	3
**Total training cost**	$5979	$5979
**TOTAL factory start‐up costs**	$188,195	$5979
**Average start‐up costs per factory**	$23,524	$747

Bouillon cube start‐up costs: Government	**2021**	**2022**
*Planning*		
Baseline survey and analyses	$249,131^a^	$50,000
Industrial assessment	$15,000	$0
Formulation of standards/norms	$20,000	$20,000
Development of monitoring and evaluation (M&E) plan	$10,000	$10,000
**Total planning cost**	$294,131	$80,000
*Equipment*		
iCheck portable fluorometer (vitamin A)	$8690	
iCheck portable photometer (iron)	$3373	
Manual centrifuge	$476	
Total number of fluorometers	12	
Total number of photometers	12	
Total number of manual centrifuges	12	
Total iCheck and manual centrifuge costs	$150,468	
Tax or duty on equipment (%)	10%	
Equipment shipping (%)	10%	
VAT (%)	8%	
**Total equipment cost**	$192,599	$0
*Social marketing/advocacy*		
Social marketing/advocacy campaign costs	$50,000	$50,000
**Total social marketing/advocacy cost**	$50,000	$50,000
*Training*		
Cost of training sessions for food regulation agency/program monitors, lab technicians, supervisors (unit cost)	$4983	$4983
Number of training sessions	5	5
**Total training cost**	$24,913	$24,913
**TOTAL government start‐up costs**	$561,643	$154,913

*Note*: Values are from the authors’ calculations. All monetary values are expressed in 2021 USD.

^a^ Some of the non‐round‐number cost estimates, here and elsewhere, are attributable to currency value adjustments made to cost estimates provided for years other than 2021.

The public sector also will face start‐up costs associated with planning, designing, and launching bouillon fortification programs. In the context of Nigeria, the bouillon fortification program is assumed to begin with a series of data collection and analysis activities to inform the design of the program, assess the status of the monitoring mechanism, and develop plans for the needed investments in government capacity. Once determined, a series of equipment investments would be made to outfit labs with the required equipment and inputs, all of which are assumed to be imported. Testing equipment that is more sophisticated than iCheck machines exists, but for some purposes, and for some micronutrients and delivery vehicles, estimates of micronutrient content can be equally reliable across machines.^33^ Regardless, more sophisticated machines are substantially more expensive to purchase, maintain, and use than iCheck machines, and hence they were not chosen for this analysis. Government is assumed to invest in social marketing to promote fortified bouillon cubes, and in the training of personnel involved in monitoring and evaluation activities (Table 6, bottom half).

### Factory operational costs: Cost calculation ingredients

Once the production of a multi‐fortified bouillon begins (in year 3 of the simulation period, by assumption), a series of operational expenses arise for industry. These are summarized in Table 7 for the first 4 years of the operational phase of the program; costs vary only slightly in subsequent years. Factories are required to continually assess product quality. Factory testing is assumed to focus on a single micronutrient (vitamin A, in this case), which would serve as a proxy for the presence of other micronutrients contained in premixes. The model can easily be adapted to allow for the testing of multiple micronutrients.

**TABLE 7:** Annual operational costs of bouillon fortification program for factories in Nigeria, 30% Codex NRVs in 2.5 g bouillon.

Annual operating costs for factories	2023	2024	2025	2026
*Fortification*				
FTE additional labor for production/premix‐related activities	1	1	1	1
Total labor cost for production/premix‐related activities	$80,750	$80,750	$80,750	$80,750
Fortification equipment maintenance (% of equipment cost)	7%	7%	7%	7%
Total equipment maintenance cost	$7022	$7022	$7022	$7022
**Total fortification cost**	$87,772	$87,772	$87,772	$87,772
*Internal quality assurance/quality control (QA/QC)*				
Portable photometer supplies—reagents for in‐house chemical analysis (unit cost per test)	$6	$6	$6	$6
Number of in‐house tests for iron per day per factory	0	0	0	0
Portable fluorometer supplies—reagents for in‐house chemical analysis (unit cost per test)	$8	$8	$8	$8
Number of in‐house tests for vitamin A per day per factory	3	3	3	3
Total reagents cost	$48,000	$48,000	$48,000	$48,000
FTE per factory for personnel for QA/QC activities	1	1	1	1
Total QA/QC personnel cost	$100,938	$100,938	$100,938	$100,938
**Total quality assurance/quality control cost**	$148,938	$148,938	$148,938	$148,938
*External quality assurance/quality control (QA/QC)*				
External lab quantitative testing (unit cost per sample tested including administration, packaging, transport/shipping, etc.)	$62	$62	$62	$62
Number of external lab tests per year per factory	12	12	12	12
**Total quality assurance/quality control cost**	$5979	$5979	$5979	$5979
*Training/retraining*				
Training/retraining for fortification and quality assurance personnel (unit cost per training session)	$249	$249	$249	$249
Number of training/retraining sessions per factory per year	1	1	1	1
**Total training/retraining cost**	$1993	$1993	$1993	$1993
*Management, overhead, administration*				
Management, overhead, administration (% of premix cost)	5%	5%	5%	5%
**Total management, overhead, administration cost**	$3,203,231	$3,282,967	$3,364,137	$3,446,749
**Total annual factory operating costs**	$3,447,914	$3,527,649	$3,608,819	$3,691,432
**Average annual operating costs per factory**	$430,989	$440,956	$451,102	$461,429

*Note*: Values are from the authors’ calculations. Management/overhead/administration costs are assumed to be a fixed percentage of total premix costs, which vary over time due to population growth. Costs in subsequent years are quite similar to those in 2026. All monetary values are expressed in 2021 USD.

Abbreviation: FTE, full‐time equivalent.

Some notable expenses relate to personnel (lab technicians, equipment operators, maintenance crews, individuals reconciling and reporting premix inventories, etc.) and reagents that are required to do in‐house testing. Factories will not likely hire additional employees to manage the new fortification tasks, but rather allocate them across existing employees. In the model, training was a small annual expense.^l^ The largest operating expense associated with fortification was the management of the purchasing (with hard currency), transporting (from international sources), the storage of and internal accounting for premix supplies, and managing the commercial issues associated with all of these increased costs. We assumed that the overall cost of management of premix supplies represented 5% of the value of premix, or roughly $3.7 million for the entire national program, or roughly $450k per factory per year for each of the eight factories in Nigeria. Premix costs for any given combination of micronutrients increase over time because of population increases, as does the costs of managing the larger flow of premix. Premix management costs will vary depending on the types and amounts of micronutrients included; less‐expensive premixes are assumed to be cheaper to manage. Premix management costs are inclusive of the internal costs to firms of engaging in policy discussions around bouillon fortification, and especially in internal discussions regarding how to deal commercially with the mandated increase in ingredient and other costs.

### Government operational costs: Cost calculation ingredients

Once the start‐up period concludes, a series of operational costs will be faced by governments. Most of these costs will recur every year; some will be constant (e.g., retraining costs), while others will vary over time due to increases in the volume of fortified products subject to government oversight. Table 8 reports (for the first 4 years of the operational phase; costs in subsequent years are quite similar to those in 2026) the activities and unit values associated with the inspections of nationally produced bouillon products and the monitoring of imported bouillon products. As is the case with factories, the majority of operational costs of inspection are personnel and management costs, with supplies required to undertake lab analyses being the second most expensive line item for monitoring both national and imported products. Note that the first 3 years of the operational period (2023–2025) are assumed to be a period of more intense monitoring and evaluation, with the number of inspections per year being reduced by 50% in 2026 and thereafter.

**TABLE 8:** Annual operational costs of bouillon fortification program for the government in Nigeria.

Annual government recurring costs	2023	2024	2025	2026
*Factory inspections and monitoring*				
Cost per factory inspection (unit cost—includes labor, travel, incidentals, management, etc.)	$162	$162	$162	$162
Number of inspections per year	96	96	96	48
Total cost of inspections	$15,546	$15,546	$15,546	$7773
Portable fluorometer supplies—reagents for sample analysis (unit cost per test)	$8	$8	$8	$8
Number of samples analyzed for vitamin A per factory inspection	5	5	5	5
Total cost of supplies for vitamin A analyses	$3840	$3840	$3840	$1920
Portable photometer supplies—reagents for sample analysis (unit cost per test)	$6	$6	$6	$6
Number of samples analyzed for iron per factory inspection	0	0	0	0
Total cost of supplies for iron analyses	$0	$0	$0	$0
Total cost of analysis of factory samples	$3840	$3840	$3840	$1920
**Total factory inspections and monitoring cost**	$19,386	$19,386	$19,386	$9693
*Import monitoring*				
FTE for import monitoring activities per port of entry	0.50	0.50	0.50	0.50
Total labor cost of import monitoring	$63,086	$63,086	$63,086	$63,086
Portable fluorometer supplies—reagents for sample analysis (unit cost per test)	$8	$8	$8	$8
Number of samples analyzed for vitamin A per consignment/delivery inspection	2	2	2	2
Total cost of supplies for vitamin A analyses	$3815	$3815	$3815	$3815
Portable photometer supplies—reagents for sample analysis (unit cost per test)	$6	$6	$6	$6
Number of samples analyzed for iron per consignment/delivery inspection	0	0	0	0
Total cost of supplies for iron analyses	$0	$0	$0	$0
Total cost of analysis of import samples	$3815	$3815	$3815	$3815
**Total import monitoring cost**	$66,901	$66,901	$66,901	$66,901
*Commercial monitoring/market surveys*				
FTE for commercial monitoring at markets/retail outlets	1	1	1	1
Total cost of labor for commercial monitoring	$12,617	$12,617	$12,617	$12,617
Portable fluorometer supplies—reagents for sample analysis (unit cost per test)	$8	$8	$8	$8
Number of samples analyzed for vitamin A per commercial outlet visit	2	2	2	2
Total cost of supplies for vitamin A analyses	$4578	$4578	$4578	$4578
Portable photometer supplies—reagents for sample analysis (unit cost per test)	$6	$6	$6	$6
Number of samples analyzed for iron per commercial visit	0	0	0	0
Total cost of supplies for iron analyses	$0	$0	$0	$0
Total cost of chemical analysis of commercial samples	$4578	$4578	$4578	$4578
**Total commercial monitoring cost**	$17,195	$17,195	$17,195	$17,195
*Household monitoring*				
Cost of new data collection or adding questions to existing annual surveys	$20,000	$20,000	$20,000	$20,000
Cost of data analysis	$10,000	$10,000	$10,000	$10,000
**Total household monitoring cost**	$0	$30,000	$0	$0
*Social marketing*				
Annual cost of social marketing, education, communication	$10,000	$10,000	$10,000	$10,000
**Total social marketing cost**	$10,000	$10,000	$10,000	$10,000
*Nutrition surveillance (impact evaluation)*				
Cost of national nutrition survey—data collection	$500,000	$500,000	$500,000	$500,000
Cost of national nutrition survey—data analysis	$50,000	$50,000	$50,000	$50,000
**Total nutrition surveillance cost**	$0	$0	$0	$0
*Training/retraining*				
Cost of training/retraining sessions for food regulation agency/program monitors, lab technicians, supervisors (unit cost)	$4983	$4983	$4983	$4983
Number of training/retraining sessions per year per factory	1	1	1	1
**Total training/retraining cost**	$39,861	$39,861	$39,861	$39,861
*Management, overhead, administration*				
Management, overhead, administration (% of monitoring cost)	5%	5%	5%	5%
**Total maintenance cost**	$5174	$6674	$5174	$4689
**Total annual government operating costs**	$158,517	$190,017	$158,517	$148,340

*Note*: Values are from the authors’ calculations. Costs in subsequent years are quite similar to those in 2026. All monetary values are expressed in 2021 USD.

Abbreviation: FTE, full‐time equivalent.

Governments are also called upon to assess the quality of fortified products in the marketplace and in households. Doing so involves the periodic collection of market and household data, and food/condiment sample collection and their analyses and reporting. Governments also engage in social marketing activities and training/retraining of public sector laboratory and other personnel as well as managing all of these processes. Finally, on occasion, governments undertake large‐scale data collection activities (e.g., nationally representative dietary intake surveys), the analyses of these data, and the reporting of policy‐relevant results.^m^ Table 8 reports the activities and unit values associated with these activities.

The reader will note that while placeholder values for national surveys and the analyses of the data that emerge from them are contained in Table 8, none has been activated in the current version of the cost model. The decision to undertake such activities periodically is important and highly recommended (modeling the addition of these activities in the cost model is trivial), but such activities are expensive and hence need to be carefully planned for.

As was assumed to be the case in factories, government testing of micronutrient content of multi‐fortified commercial products is pegged on vitamin A,^n^ for which a quantitative rapid test exists, and assuming that confirmation of this single micronutrient (plus the review of premix certification documents) will be sufficient to assess the degree of overall compliance with fortification standards. Premixes containing multiple micronutrients may require additional testing and associated increases in laboratory and other costs; this is easily accommodated in the cost model. Shifting a focus to testing another micronutrient, such as iron, would have minimal impacts on costs, but the model could easily be adjusted to address these new costs.

### Summary estimates of national bouillon fortification costs^o^^,^ p

Table 9 begins by reporting population sizes, a key driver of program costs. Clearly, the Nigerian population is substantially larger than those of Senegal and Burkina Faso. Moreover, bouillon consumption patterns also differ considerably across countries (see Table 2). Therefore, in the context of Nigeria, population size and high relative rates of bouillon consumption combine to generate very large premix requirements (which represent roughly 95% of program costs, even with the program operating at 75% compliance) and overall program costs (Table 9, total cost and premix percentage data lines).

**TABLE 9:** Summary estimates of bouillon fortification program costs for Senegal, Burkina Faso, and Nigeria, 30% of Codex NRVs in 2.5 g bouillon.

Summary cost measures	Senegal	Burkina Faso	Nigeria
Population size (2021)^a^	17,196,308	21,497,097	211,400,704
Bouillon‐consuming population (2021)	15,395,312	17,633,742	207,922,676
Total costs ^b^			
Total 10‐year costs	$27,689,836	$20,770,738	$642,733,105
Total annual average cost (over 10 years)	$2,768,984	$2,077,074	$64,273,311
Total start‐up costs (average annual cost over 2 years)	$649,952 ($324,976)	$647,595 ($323,798)	$910,730 ($455,365)
Total nonpremix operational costs (average annual cost over 8 years)	$1,013,971 ($126,746)	$863,669 ($107,959)	$31,207,812 ($3,900,977)
Total premix costs (annual average costs over 8 years)	$26,025,914 ($3,253,239)	$19,259,475 ($2,407,434)	$610,614,563 ($76,326,820)
Unit costs			
Cost per MT of fortified bouillon^c^	$319	$323	$333
Cost per bouillon consumer reached	$0.20	$0.13	$0.34
Cost shares (%)			
Government (start‐up phase; nonpremix, operational phase)	4% (88%, 46%)	5% (90%, 50%)	0.3% (79%, 4%)
Industry (start‐up phase; nonpremix, operational phase)	2% (12%, 54%)	2% (10%, 50%)	4.7% (21%, 96%)
Premix^d^ (only, as a % of total cost)	94%	93%	95%
Sources of bouillon (%)			
Domestic production^e^	11%	11%	92%
Imported	89%	89%	8%
Product requirements (8‐year annual average)			
Fortified bouillon (MT/year)	10,861	8037	240,925
Premix (MT/year)	176	131	3915

*Note*: Values are from the authors’ calculations. All monetary values are expressed in 2021 USD.

Abbreviation: MT, metric tons.

^a^ Population data are taken from the United Nations World Population Prospects, 2019 update.^31^

^b^ The hypothetical bouillon fortification program envisioned for this costing exercise delivers bouillon fortified with five micronutrients (vitamin A, folic acid, B12, iron, and zinc) at 30% of Codex Nutritional Reference Value for women of reproductive age, assuming a 2.5 g/day serving size. Iodine is also included in the fortified cube, but it is not included in cost calculations since it is assumed to be delivered by iodized salt, an ingredient not included in our cost model, which focuses only on marginal costs of fortification.

^c^ Slight differences across countries in cost per MT are attributable to differences in wage rates and import duties.

^d^ Ultimately, the bulk of premix costs will be paid by consumers, for both nationally produced and imported products.

^e^ These percentages are slightly different from the 10% domestic/90% imported production source assumptions reported in the text; percentages reported here reflect premix costs for the entire program, which include differences in import duties on imported bouillon versus imported premixes.

Total program costs, and the start‐up, operational, and premix costs that comprise them are reported in the next set of rows of Table 9; all costs are first reported as total costs over their respective time periods (total program costs, 10 years; start‐up costs, 2 years; operational and premix costs, 8 years) and as annual averages over their respective time periods. Burkina Faso faces the lowest costs across all cost components, followed by Senegal; Nigeria has the most expensive program in absolute terms, by far.

Since the premix formula is identical for each country (30% of Codex NRV for vitamin A, iron, folic acid, vitamin B12, and zinc in 2.5 g of bouillion) and the costs of fortificants are assumed to be the same for all study countries and for countries exporting to them, the cost per MT of fortified bouillon is roughly the same for each country (∼$325); small differences are attributable to cross‐country differences in premix management costs at the factory level. However, because the proportion of the population reached by bouillon in each country varies and mainly because the average quantities of bouillon consumption differ across countries (Burkina Faso, 1.45 g/WRA/day; Senegal, 2.3 g/WRA/day; Nigeria, 3.65 g/WRA/day), the cost per consumer reached is much higher in Nigeria ($0.34) than for Senegal ($0.20) or for Burkina Faso ($0.13).

Cost shares vary across stakeholders (government, industry, consumers) and between the start‐up and operational phases. These patterns vary across countries primarily because of the heavy reliance in Senegal and Burkina Faso on imported bouillon cubes (see Tables S1 and S2). Senegal, and especially Burkina Faso, have far fewer in‐country factories so total up‐front and recurring fortification costs of national production are lower than is the case for Nigeria. Once again, in all countries, premix costs represent the largest proportion of total costs, by far.

The final set of rows of Table 9 report the amounts of fortified bouillon needed to meet national demand and the required amount of premix; once again, the results are driven mainly by population size and bouillon consumption patterns, with ∼241k MT per year of fortified bouillon required by Nigeria, and only small fractions of that sum required to meet the needs of consumers in Senegal and Burkina Faso. This is important because there are costs associated with managing the flows of premix into countries; in Nigeria, the scale of this flow makes it challenging and expensive to manage.

### Estimates of national bouillon fortification costs, by program phases and cost categories

**FIGURE 2:**
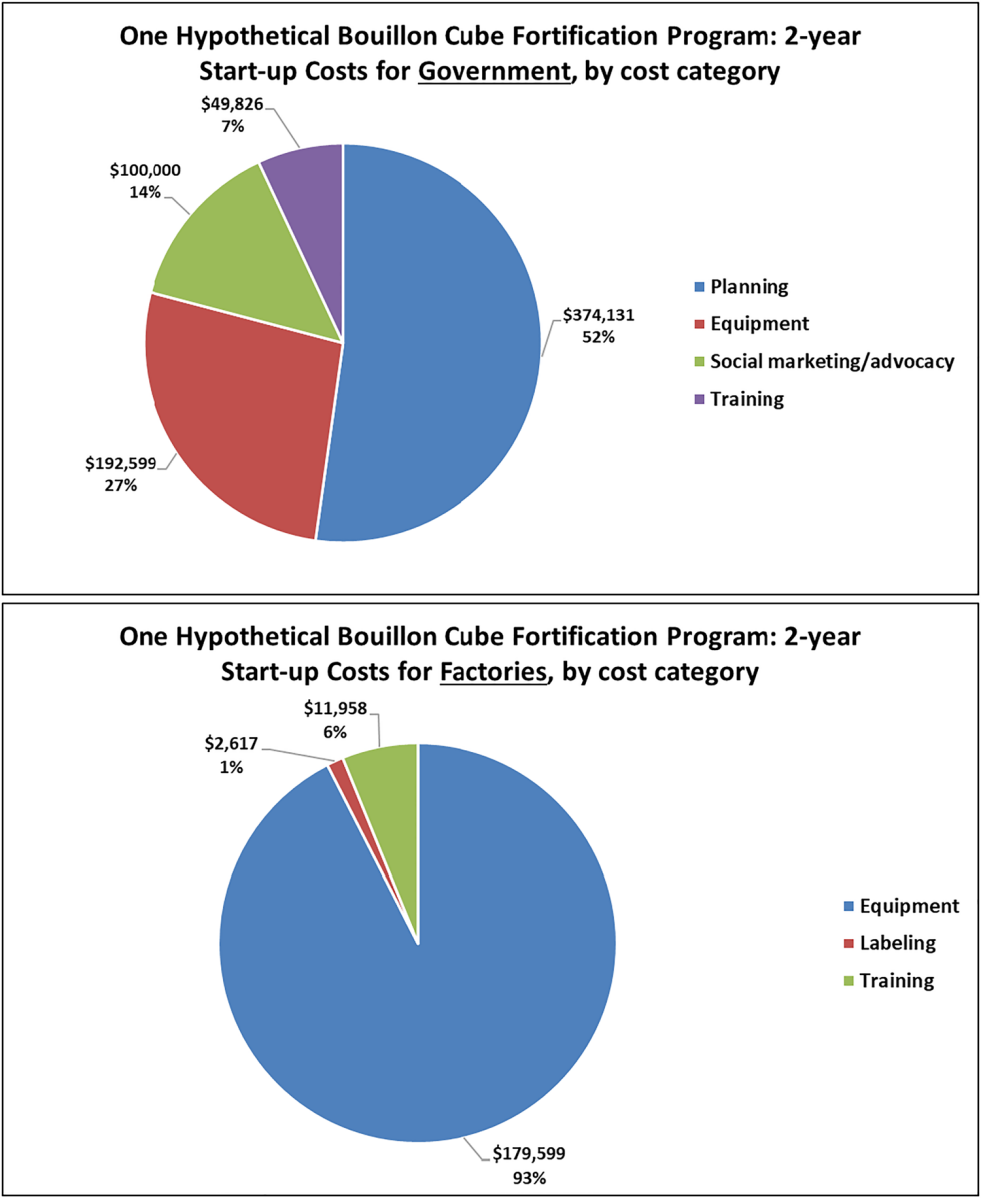
Government and factory start‐up costs of bouillon fortification by cost category for Nigeria, 30% of Codex NRVs in 2.5 g bouillon. *Note*: Values are from the authors’ calculations. All monetary values are expressed in 2021 USD.

Finally, we provide a more granular look at the composition of costs during the start‐up and operational phases for industry and for governments, and a 1‐year snapshot of fortification program costs at the mid‐point of the model simulation period to remind readers of the cost allocation decisions that must be taken. We present results for Nigeria, but similar patterns (though at very different scales) are present in the cases of Senegal and Burkina Faso (see the Supplementary Material). Figure 2 reports the bouillon fortification program start‐up costs for the government in Nigeria. Planning and the purchase and installation of required testing equipment comprise nearly 80% of all start‐up costs. Social marketing and training costs are also faced.

Compared to the government, factories (Figure 2) spend much more heavily (proportionately) on the purchase and installment of new equipment (production line as well as laboratory equipment), face some training costs, and invest in designing and producing new product labels.^q^ In the MINIMOD cost model, import monitoring costs are not linked to the volume of bouillon cubes coming across borders. Rather, in the Nigerian context, these costs account for the personnel at each of the 10 ports of entry (0.5 full‐time equivalents per port), and the testing of products (25 deliveries tested per year per port of entry). Salaries sum to approximately $63,000 per year, with estimated testing costs of approximately $3800 per year.

**FIGURE 3:**
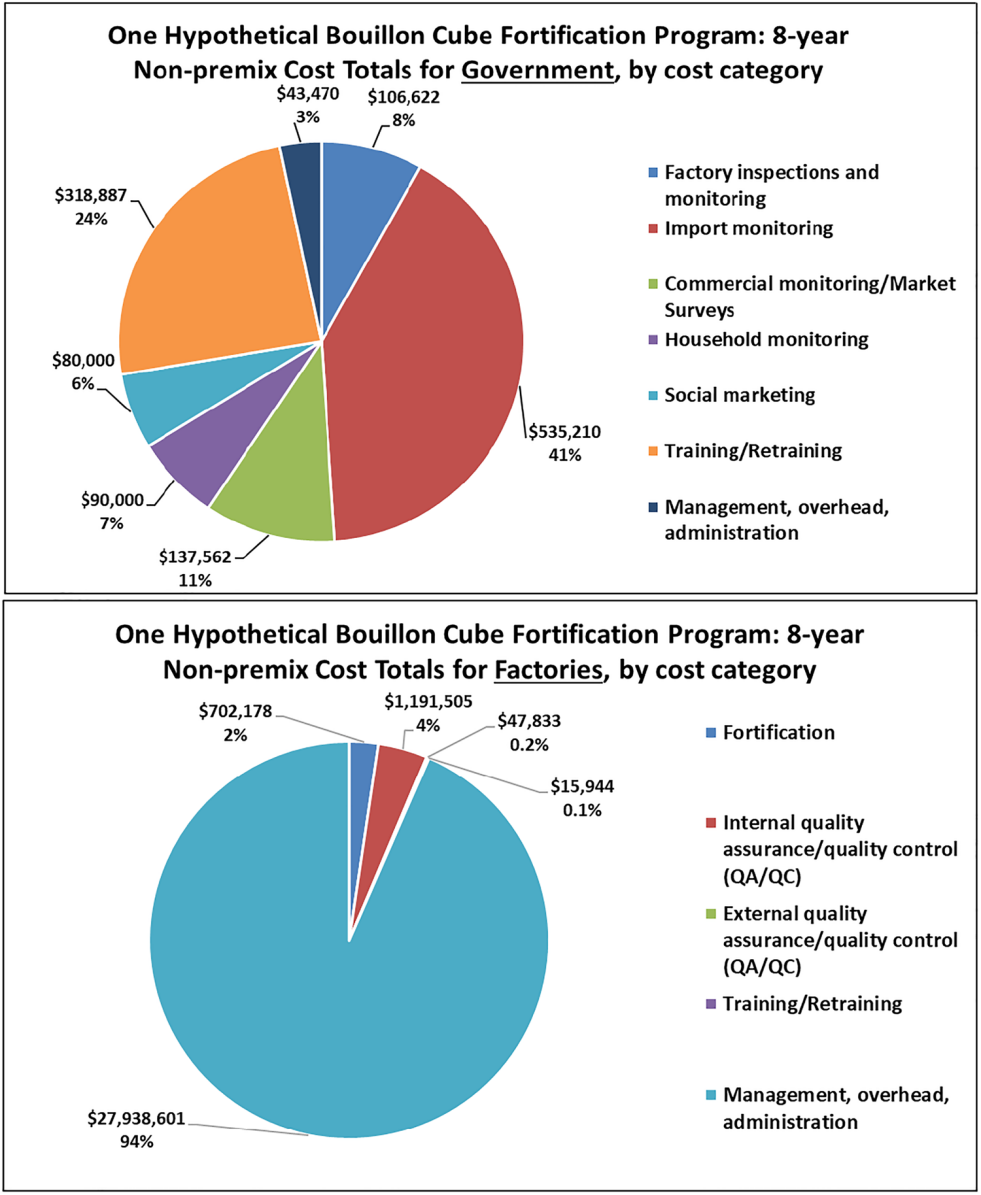
Government and factory nonpremix operational costs of bouillon fortification by cost category for Nigeria, 30% of Codex NRVs in 2.5 g bouillon. *Note*: Values are from the authors’ calculations. All monetary values are expressed in 2021 USD.

Once the bouillon fortification program is fully operational (in year 3 of the simulation period, by assumption), an array of costs will be faced by governments (Figure 3). The monitoring and control of imported products represents ∼40% of operational costs; although 90% of bouillon consumed in Nigeria is produced in Nigeria, the remaining 10% is imported and must be monitored to ensure that it meets the same fortification standards that domestically produced bouillon must meet. Monitoring the eight national production facilities is much less costly. Periodic training/retraining of personnel involved in monitoring national and imported bouillon products requires substantial costs (∼$320k over 8 years). Social marketing and the collection and analyses of marketplace and household data comprise the remaining important operational cost categories.

The operational costs faced by industry (Figure 3) are highly skewed toward the management of the overall fortification process, including (most notably) the procurement (including securing hard currency for international procurement), international transportation, customs clearing, national transportation, and storage of the very large amounts of premix required by this hypothetical fortification program; by assumption, this represents 5% of the total value of the imported premix. All premixes are assumed to be imported. The 5% premix‐value‐based estimate (derived from discussions with industry representatives and experts in the field) of the management, overhead, and administration costs of industry fortification reflects participation in national discussions of the design and regulation of fortification programs, the cost of managing premix supply chains (e.g., securing foreign exchange, managing premix transportation, importation, storage, premix inventory maintenance, and reconciliation, etc.), and the costs associated with passing premix costs along to consumers in the form of higher retail prices in a highly competitive industry. This 5% assumption will not affect the relative costs or cost‐effectiveness of different premix options. The costs of the fortification process itself, which is essentially automatically done by specialized machinery, are low; the costs of internal quality control activities are somewhat larger.

**FIGURE 4:**
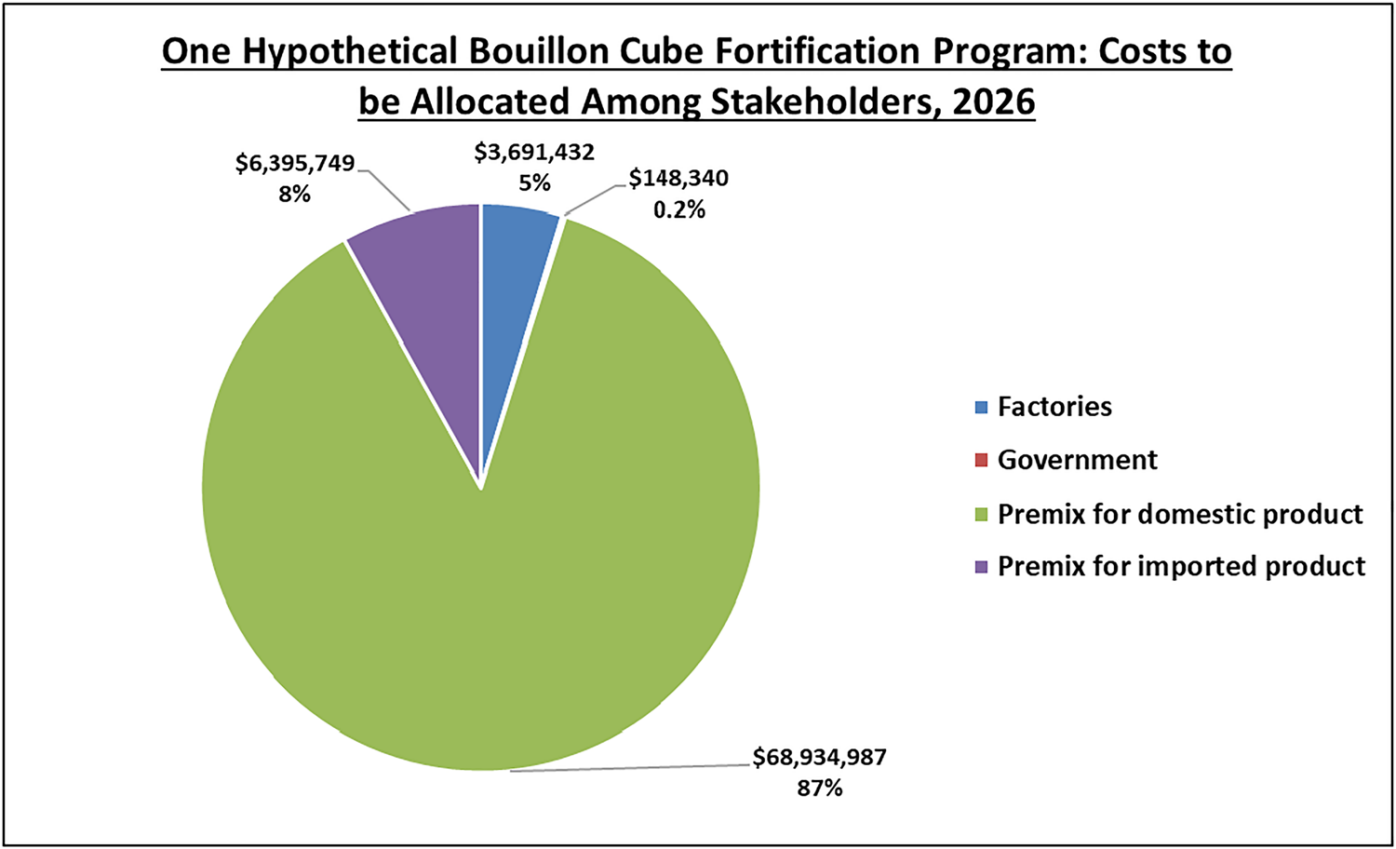
Operational year costs of bouillon fortification by cost category, 2026 for Nigeria, 30% of Codex NRVs in 2.5 g bouillon. *Note*: Values are from the authors’ calculations. All monetary values are expressed in 2021 USD.

Finally, Figure 4 provides a summary snapshot of the total annual costs associated with a fortified bouillon program at the mid‐point of the operational period, by four categories that roughly align with stakeholder groups involved in or affected by the program: factories, government, domestic premix, and premix costs associated with imported products. Roughly 87% of total annual costs (∼$68 m) are attributable to the cost of purchasing and managing the premix required to fortify nationally produced bouillon cubes. Traditionally, these costs are passed on to consumers in the form of higher retail prices for fortified bouillon cubes, which is reasonable since consumers are the direct beneficiaries of fortification programs. However, in the context of Nigeria, the volume of costs that must be passed along to consumers is very large and may be challenging to accomplish in highly competitive markets.

## DISCUSSION

Some decision‐makers aiming to address inadequate intake of micronutrients in LMICs are considering bouillon cubes as a vehicle for delivering micronutrients to at‐risk populations. The extensive geographic and socioeconomic reach of this condiment vehicle has been demonstrated in Nigeria, Senegal, and Burkina Faso, and evidence on the potential nutritional and child‐mortality–reducing benefits in these countries is emerging.^9^, ^10^, ^11^, ^14^ The final pieces of evidence required to fully assess the wisdom of investing in the design and implementation of a multi‐fortified bouillon cube are technical feasibility, commercial viability, and (related) program cost. This paper addressed the program cost issue for specific hypothetical multi‐fortified bouillon programs in each country, paying close attention to premix costs (which consumers usually pay but which industry must manage) and to the stakeholders likely required to cover specific types of costs.

The issue of cost in the context of LSFF programs is not new.^25^, ^37^, ^38^, ^39^, ^40^, ^41^, ^42^ Indeed, activity‐based accounting has been an established practice for over 50 years.^43^ Approaches within the domain of activity‐based accounting of LSFF programs have varied (e.g., some authors take a social perspective,^39^ while others focus on private costs^42^), some authors take single annual snapshots of costs,^44^ while others take a broader temporal approach that includes start‐up as well as operational costs.^35^ That said, this paper makes several contributions to the existing literature in that it: (a) explicitly addresses the start‐up costs associated with planning and launching new LSFF programs; (b) identifies three sets of stakeholders (governments, firms, consumers) and the intervention programs’ costs that each may be called upon to pay over time; and (c) allows for flexibility in addressing the costs of alternative micronutrients and quantities of each (premix cost calculator).

We found that start‐up costs associated with designing and preparing to implement a bouillon fortification program can be substantial, both for governments and for industry. In the context of Nigeria, government and industry costs over a 2‐year start‐up period were ∼$717k and ∼$194k, respectively. These costs were lower in the cases of Burkina Faso and Senegal (∼$566k and ∼$66k for Burkina Faso and ∼$573k and ∼$76k for Senegal, for the government and industry, respectively; for governments, this cost difference was driven mainly by the number of firms in the industry). In all cases, some components of the start‐up costs were considerable (e.g., planning costs for governments and equipment costs for industry), while others were relatively small (e.g., training costs for governments and for industry). However, careful attention should be paid to even the relatively small costs since a failure to pay them can compromise the efficiency and (ultimately) the sustainability of the entire program. The allocation of costs can shift considerably during the operational phase of the fortification program, but the extent of these shifts will depend on the scale of the program and on the proportion of the total bouillon supply that is produced nationally. For example, in the case of Nigeria, government costs became small relative to industry costs, and both of these (combined) were dwarfed by premix costs that would likely be passed to consumers in the form of increased bouillon product costs. Industry will be responsible for managing the flows of premix into and through production facilities, and for conveying these costs to consumers, both of which are challenging tasks, especially in competitive markets. At the other extreme, in Burkina Faso, where currently no bouillon is produced and only 10% of all bouillon consumed in the country is assumed to be produced in the country over the modeling time horizon, premix imports will be limited as will the costs faced by the national industry. In this case, consumers will pay (to foreign producers) higher prices for fortified bouillon products.

In model simulation results not presented here, it was clear that while government costs and industry nonpremix costs varied across countries, these costs tended to be stable regardless of the number, types, and amounts of fortificants included in the bouillon fortification program. The same is not true, however, for premix costs that can vary dramatically depending on choices (embodied in fortification standards) made regarding the content of premixes. National standards will be greatly influenced by national dietary needs and levels of bouillon consumption; regional choices will consider nutritional needs and levels of bouillon consumption at more aggregate levels, with consequences for national benefits and costs. Regardless of the geo‐political scale at which standards are set and while all costs are important, premix costs in particular should be considered when designing bouillon fortification programs as they may determine program design decisions, with consequences for the public health impact.

Finally, national production versus importation of fortified products is a perennial issue. One of the key advantages of national production is that a country can develop and implement a national fortification standard. Countries with no national production (e.g., Burkina Faso, currently) may essentially be forced to adopt the bouillon fortification standards of producing/exporting countries. Ongoing regional harmonization discussions related to bouillon fortification seek to address this issue. From the perspective of fortification program costs, the implications of shifting from importation to national production of bouillon are not large. For example, for governments, managing imported bouillon products tends to be about three times more expensive than managing national production. However, these cost differences are very small compared to the main driver of fortification programs costs (premix costs) which does not change with the proportion of product nationally produced since all bouillon products, regardless of source, are required to be fortified at the same levels with the same micronutrients.

The research reported in this paper has several limitations. First, the multi‐fortified bouillon programs examined here are hypothetical. While some voluntary fortification programs exist, for example, some Nestlé bouillon cubes contain iron (in addition to iodine),^45^ these cubes contain limited amounts of single micronutrients. That said, the technical feasibility of introducing all five of the micronutrients envisioned in this hypothetical program, and in greater quantities than assumed here, has successfully been tested and found to be acceptable to consumers in northern Ghana.^21^ Second, our focus was exclusively on large‐scale bouillon production facilities; circumstances relevant to costing analysis might be different for smaller‐scale operations. However, large‐scale operations tend to dominate bouillon markets in the countries included in this study, even in rural areas (e.g., the top four brands in Nigeria represent ∼80% of the market),^18^, ^32^ all of these are large‐scale operations. Third, fortificant prices, which are known to vary seasonally and otherwise, are held constant over the entire simulation time period. There are no comprehensive time series data on fortificant prices, and fortificant quality issues (which are generally not observable) make price differences at any point in time very difficult to interpret. To compensate for this possible shortcoming, to the extent possible, we undertook sensitivity analyses based on possible differences in fortificant prices and reported those results in the Supplementary Material. Fourth, we assumed that 75% of bouillon cubes would be fortified at 100% of the standard beginning at the outset of the operational phase of the program (year 3 of the 10‐year model simulation period). Implementation of the program might be more gradual and/or less complete than we assume, and the patterns and ultimate levels of industry compliance in each country are unknown. Both the timing and the level of compliance can affect program costs, for example, low levels of compliance can be linked to low levels of spending on monitoring and evaluation of both in‐country bouillon production and bouillon imports. That said, the gradual appearance of fortified bouillon cubes in the market would reduce costs (less premix would be required, in particular) and the same would be true if adherence to standards were lower or slower to develop, or both. Moreover, all of these factors could vary depending on whether fortification standards are voluntary or mandatory. Fifth, the cost analysis dealt with the degradation of fortificants (vitamins only) by adjusting premix formulas to account for degradation via overages; our assumed overage rates might not be precise. Finally, and generally, assumptions had to be made in constructing each of the country cost models. We list these explicitly in Table 1 and the associated text, and we relied heavily on in‐country collaborators to generate, review, and confirm all modeling assumptions.

## CONCLUSIONS

Next to salt, bouillon cubes are the most commonly consumed condiment in much of West Africa, and their reach extends to all socioeconomic groups, including the rural poor.^35^ The commercial viability for bouillon cubes to convey small amounts of individual micronutrients, iron and vitamin A, in particular, has already been demonstrated. Some of the technical challenges associated with including more and larger quantities of micronutrients have been solved in a scientific context, although addressing these and other challenges in commercial contexts remains to be done. The remaining issues are which micronutrients and how much of each to include in a multi‐fortified bouillon cube. The potential contributions of multi‐fortified bouillon cubes to reducing inadequate intake of vitamin A, folate, vitamin B12, iron, and zinc have been demonstrated,^9^, ^10^, ^11^ as have the potential child‐life‐saving benefits of achieving dietary adequacy in vitamin A, folate, and zinc.^14^ Therefore, bouillon is an excellent candidate delivery vehicle for reducing micronutrient inadequacies in West Africa. This paper addressed the issue of bouillon fortification program costs.

Bouillon fortification programs will require resources to establish and to maintain. Different stakeholders will be called upon to pay different program costs; deciding on cost shares should be part of the policymaking process and hence will play out differently in each country context. More specifically, governments, perhaps with assistance from international stakeholders, will likely pay the majority of start‐up costs, including social marketing costs and those associated with establishing the monitoring and evaluation framework that will be required to maintain a level commercial playing field in the bouillon cube market. During the early years of program operation, as industry adapts to higher and different costs, and to the challenges of managing new supply chains, it is hard to predict which stakeholder groups will cover which fortification program costs, for example, governments may temporarily (at least) reduce import duties and value‐added taxes on imported premixes to facilitate adherence to standards. However, if bouillon markets are competitive, in the end, consumers will pay the bulk of the cost associated with fortification via increases in the prices of fortified bouillon cubes, which is reasonable given that the benefits of bouillon fortification will accrue mainly to consumers at one or more points along the life cycle (e.g., child survival, improved school performance, better‐paying jobs, etc.).

The levels of program costs will be greatly influenced by population size and average daily consumption of bouillon cubes. The composition of costs will be influenced by the proportion of bouillon cubes that are nationally produced versus imported.

As with all large‐scale fortification programs, premix costs represent the largest share of overall program costs, but the absolute size of premix costs will depend on which fortificants are included and at what levels. Some fortificants will be included in relatively small amounts and are inexpensive (e.g., folic acid), while others will likely be included in larger amounts and are relatively more expensive (e.g., vitamin A). All increases in premix costs will put upward pressure on retail prices of bouillon cubes; this process is commercially challenging and may also reduce demand among the lowest socioeconomic groups.

Although the largest share of program costs will be premix costs (almost regardless of the premix formula chosen), small costs matter greatly. For example, failure to pay government monitoring and evaluation costs can undermine the level playing field in the marketplace, thereby compromising the sustainability of the fortification program. Or, failure to invest in social marketing might reduce demand for fortified bouillon cubes, thereby reducing the program's public nutritional and health benefits.

Program costs should be estimated (tools exist for doing so) during the design phase and woven into stakeholder discussions; knowing these costs and weaving them into program choice and program design discussions will enhance the efficiency and sustainability of the programs ultimately chosen. Program costs should always be set alongside estimates of programs’ nutritional and other benefits in policy discussions.

## AUTHOR CONTRIBUTIONS

S.A.V., M.J., and K.P.A. designed the research and developed the country‐specific model templates. O.M.A., R.E.‐S., M.B., F.I., K.K., B.O., and H.S. provided country‐specific model parameters. S.A.V., M.J., and K.P.A. prepared the first draft of the paper. All authors reviewed several drafts of the paper and approved the final version.

## COMPETING INTERESTS

All authors report no conflicts of interest.

### Peer review

The peer review history for this article is available at https://publons.com/publon/10.1111/nyas.15234.

## Supporting information



Supporting Information

## Footnotes

a At the time of publication, no bouillon cubes were being produced in Burkina Faso, although many were being sold there.^18^ However, a 10‐year cost model envisioning domestic production contributing 10% of total national supply was developed for Burkina Faso to inform stakeholders. Parameters in the operational portion of the cost model were modified to capture the reality of a program relying heavily on imported multi‐fortified cubes over the entire simulation period.

b No discount rate is applied.

c These activities are comprised of sets of inputs (labor, materials, etc.) that also have unit prices; these ingredients to activities are suppressed in the equations presented here, but are explicitly addressed in the results section, below.

d For simplicity, the various governmental institutions and the consultants hired to assist them that generally undertake public sector activities associated with the management and regulation of LSFF programs are collectively labeled “government” in this costing exercise. No activities are excluded, regardless of the actors involved in planning and implementing them.

e Activity labels may be similar for firms and government (e.g., training), but the types of training, the individuals involved, and the prices of these activities can and do vary.

f Frequency and amounts of bouillon consumption are taken from Adams et al. In the case of Nigeria, recent evidence suggests that bouillon consumption might be somewhat higher than values reported here and used in our cost calculations. ^9^, ^10^, ^11^

g As of January 2024, 100% of bouillon cubes consumed in Burkina Faso are imported. The cost model simulation period extends to 2030; by the end of that period, we expect some national bouillon production in Burkina Faso. That said, the implications for total cost of national production versus imported cubes are small.

h More specifically, the following fortification levels (in terms of mg/kg of bouillon) apply to this case: iron (2640 mg/kg), vitamin A (96 mg/kg), zinc (1680 mg/kg), vitamin B12 (0.288 mg/kg), and folic acid (28.8 mg/kg). Note that costs are sensitive to the fortificants chosen for each micronutrient. For example, micronized ferric pyrophosphate (FePP) was chosen as the iron fortificant for all results presented here; if electrolytic iron were chosen, the costs would be substantially lower.

i Real fortificant prices were based on expert consultation with individuals active in the micronutrient fortificant market, and represent market prices as of July 6, 2023.

j It was announced at the December 6, 2023 meeting of the Nigerian National Fortification Alliance that the import duties on premixes will be reduced from 20% to 5%. In the cost model, this reduction in import duties would lead to a reduction in average annual premix costs for domestic bouillon production from ∼$70m to ∼$61m.

k Imported fortified oils, flours, and condiments are taxed upon import at a rate commensurate with their respective food categories; we do not include in our cost calculations the increase in tax attributable to the (now) higher price of the fortified product.

l Losses due to product and/or premix spoilage or waste were not included in the cost analysis.

m Broad‐based data collection activities are assumed to take place every 5 years and costs are assumed to be distributed across all the programs that would benefit from the evidence that they generate. Here, we assume that $500k of data collection costs and $50k of data analysis/reporting costs would be included in the budgets for the fortified bouillon program.

n Vitamin A is known to degrade with exposure to light, heat, and humidity^34^ and, therefore, requires that overages be added to compensate for degradation. Overage rates are included explicitly in all standards for many vitamins; testing thresholds at factories would consider these thresholds.

o Estimates of subnational nutritional *benefits* of LSFF programs are available^9^, ^10^, ^11^ and can be linked to subnational estimates of LSFF program costs,^35^ but given the very extensive reach of bouillon cubes spatially and across socioeconomic groups and their relatively uniform consumption across households, we present only national results of program costs here.

p For proper interpretation and use in policy discussions, the expected streams of LSFF program costs must align with program benefits, temporally and spatially. See Vosti et al. 2024 for details of how this was achieved in the context of this research.^15^

q It is assumed that factories will follow the regulations that require the reporting of the micronutrient content of their products and take advantage of regulations permitting the advertising (on labels) of micronutrient content.
