# Supplementary material for: The costs and the potential allocation of costs of bouillon fortification: The cases of Nigeria, Senegal, and Burkina Faso
Source: Ann N Y Acad Sci. 2024 Oct 21;1541(1):181–201. doi: 10.1111/nyas.15234 (PMC11580765; doi:10.1111/nyas.15234)
Supplement: Supplementary file 1 — Supporting Information [file NYAS-1541-181-s001.docx]

**Supplementary Table 1: Structure and Characteristics of the Bouillon Industry, Senegal**

| Bouillon cube industry information |  |
| --- | --- |
| Number of bouillon cube factories | 3 |
| Average number of days of bouillon cube production per factory per year | 250 |
| Average wage of factory labor employee ($/year) | $3,978 |
| Average wage of factory lab or quality assurance employee ($/year) | $4,972 |
|  |  |
| Government monitoring and evaluation (M&E) information |  |
| Number of industrial scale bouillon cube factories | 3 |
| Number of on-site factory inspections (per factory per year) | 12 |
| Number of imported bouillon cube ports of entry | 2 |
| Number of imported bouillon cube consignments/deliveries to be inspected (per port of entry per year) | 25 |
| Number of commercial site visits for commercial monitoring (total number per monitoring year) | 300 |
| Average wage of import monitor (USD/year) | $4,972 |
| Average wage of commercial monitor (USD/year) | $4,972 |
|  |  |
| Structure of consumption |  |
| % of total national consumption that is produced domestically | 10% |
| % of total domestic production that is fortifiable/industrially produced | 100% |
| % of total national consumption that is imported | 90% |
| % of total imports that is fortifiable/industrially produced | 100% |

Source: Authors’ calculations. All monetary values are expressed in 2021 USD.

**Supplementary Table 2: Structure and Characteristics of the Bouillon Industry, Burkina Faso**

| Bouillon cube industry information |  |
| --- | --- |
| Number of bouillon cube factories | 3 |
| Average number of days of bouillon cube production per factory per year | 250 |
| Average wage of factory labor employee ($/year) | $2,516 |
| Average wage of factory lab or quality assurance employee ($/year) | $3,144 |
|  |  |
| Government monitoring and evaluation (M&E) information |  |
| Number of industrial scale bouillon cube factories | 3 |
| Number of on-site factory inspections (per factory per year) | 12 |
| Number of imported bouillon cube ports of entry | 2 |
| Number of imported bouillon cube consignments/deliveries to be inspected (per port of entry per year) | 25 |
| Number of commercial site visits for commercial monitoring (total number per monitoring year) | 300 |
| Average wage of import monitor (USD/year) | $3,144 |
| Average wage of commercial monitor (USD/year) | $3,144 |
|  |  |
| Structure of consumption |  |
| % of total national consumption that is produced domestically | 10% |
| % of total domestic production that is fortifiable/industrially produced | 100% |
| % of total national consumption that is imported | 90% |
| % of total imports that is fortifiable/industrially produced | 100% |

Source: Authors’ calculations. All monetary values are expressed in 2021 USD.

**Supplementary Table 3: Premix Costs for Domestic Production of Multiple Micronutrient-Fortified Bouillon from 2023-2030, by Cost Category, Senegal**

| **Premix for domestic product** | **2023** | **2024** | **2025** | **2026** | **2027** | **2028** | **2029** | **2030** |
| --- | --- | --- | --- | --- | --- | --- | --- | --- |
| ***Premix for domestic food vehicle*** |  |  |  |  |  |  |  |  |
| Availability of bouillon cubes in food supply (g/capita/day) | 2.00 | 2.00 | 2.00 | 2.00 | 2.00 | 2.00 | 2.00 | 2.00 |
| Total population | 18,116,526 | 18,585,815 | 19,062,244 | 19,545,826 | 20,036,323 | 20,533,936 | 21,038,921 | 21,551,461 |
| Annual volume of food vehicle (MT) | 13,244 | 13,587 | 13,935 | 14,289 | 14,648 | 15,011 | 15,380 | 15,755 |
| % of national consumption that is produced domestically | 10% | 10% | 10% | 10% | 10% | 10% | 10% | 10% |
| % of total domestic production that is **fortifiable/industrially produced** | 100% | 100% | 100% | 100% | 100% | 100% | 100% | 100% |
| % of **fortifiable** domestic product that is **fortified** | 75% | 75% | 75% | 75% | 75% | 75% | 75% | 75% |
| Annual volume of fortified food vehicle (MT) | 993 | 1,019 | 1,045 | 1,072 | 1,099 | 1,126 | 1,154 | 1,182 |
| Transportation/distribution/storage ($/kg) | 0.20 | 0.20 | 0.20 | 0.20 | 0.20 | 0.20 | 0.20 | 0.20 |
| Tax or duty (%) | 25% | 25% | 25% | 25% | 25% | 25% | 25% | 25% |
| Cost per kg of premix | $20.64 | $20.64 | $20.64 | $20.64 | $20.64 | $20.64 | $20.64 | $20.64 |
| Premix cost per MT of cubes, including shipping, taxes, etc. | $335.41 | $335.41 | $335.41 | $335.41 | $335.41 | $335.41 | $335.41 | $335.41 |
| Total cost of premix for domestic food vehicle | $333,163 | $341,793 | $350,555 | $359,448 | $368,468 | $377,619 | $386,906 | $396,332 |

Source: Authors’ calculations. All monetary values are expressed in 2021 USD.

**Supplementary Table 4: Premix Costs for Domestic Production of Multiple Micronutrient-Fortified Bouillon from 2023-2030, by Cost Category, Burkina Faso**

| **Premix for domestic product** | **2023** | **2024** | **2025** | **2026** | **2027** | **2028** | **2029** | **2030** |
| --- | --- | --- | --- | --- | --- | --- | --- | --- |
| ***Premix for domestic food vehicle*** |  |  |  |  |  |  |  |  |
| Availability of bouillon cubes in food supply (g/capita/day) | 1.17 | 1.17 | 1.17 | 1.17 | 1.17 | 1.17 | 1.17 | 1.17 |
| Total population | 22,720,842 | 23,351,513 | 23,995,149 | 24,651,776 | 25,321,196 | 26,003,242 | 26,697,615 | 27,404,036 |
| Annual volume of food vehicle (MT) | 9,732 | 10,002 | 10,278 | 10,559 | 10,846 | 11,138 | 11,436 | 11,738 |
| % of national consumption that is produced domestically | 10% | 10% | 10% | 10% | 10% | 10% | 10% | 10% |
| % of total domestic production that is **fortifiable/industrially produced** | 100% | 100% | 100% | 100% | 100% | 100% | 100% | 100% |
| % of **fortifiable** domestic product that is **fortified** | 75% | 75% | 75% | 75% | 75% | 75% | 75% | 75% |
| Annual volume of fortified food vehicle (MT) | 730 | 750 | 771 | 792 | 813 | 835 | 858 | 880 |
| Transportation/distribution/storage ($/kg) | 0.20 | 0.20 | 0.20 | 0.20 | 0.20 | 0.20 | 0.20 | 0.20 |
| Tax or duty (%) | 25% | 25% | 25% | 25% | 25% | 25% | 25% | 25% |
| Cost per kg of premix | $20.64 | $20.64 | $20.64 | $20.64 | $20.64 | $20.64 | $20.64 | $20.64 |
| Premix cost per MT of cubes, including shipping, taxes, etc. | $335.41 | $335.41 | $335.41 | $335.41 | $335.41 | $335.41 | $335.41 | $335.41 |
| Total premix cost for domestic food vehicle | $244,821 | $251,617 | $258,552 | $265,628 | $272,841 | $280,190 | $287,672 | $295,284 |

Source: Authors’ calculations. All monetary values are expressed in 2021 USD.

**Supplementary Table 5: Bouillon Fortification Start-up Costs for Factories, Senegal**

| Bouillon cube factory startup costs | 2021 | 2022 |
| --- | --- | --- |
| *Equipment* |  |  |
| Feeder (unit cost) and installation | $5,000 |  |
| Number of factories requiring a feeder | 3 |  |
| Total feeder cost | $15,000 |  |
| iCheck portable fluorometer | $8,690 |  |
| iCheck portable photometer | $3,373 |  |
| Manual centrifuge | $476 |  |
| Number of factories requiring a fluorometer | 3 |  |
| Number of factories requiring a photometer | 3 |  |
| Number of factories requiring a centrifuge | 3 |  |
| Total fluorometer, photometer, and centrifuge costs | $37,617 |  |
| Tax or duty on factory equipment (%) | 10% |  |
| Equipment shipping (%) | 10% |  |
| VAT (%) | 15% |  |
| Total equipment cost | $71,033 |  |
| *Labeling* |  |  |
| Redesign of labels and label printing plates (unit cost) | $327 |  |
| Number of factories/brands requiring label and printing plates redesign | 3 |  |
| Total labeling cost | $981 |  |
| *Training* |  |  |
| Training and sensitization for fortification and quality assurance personnel (unit cost per training session) | $249 | $249 |
| Number of training sessions per factory | 3 | 3 |
| Total training cost | $2,242 | $2,242 |
| TOTAL factory start-up costs | $74,256 | $2,242 |
| Average start-up costs per factory | $24,752 | $747 |

Source: Authors’ calculations. All monetary values are expressed in 2021 USD.

**Supplementary Table 6: Bouillon Fortification Start-up Costs for Factories, Burkina Faso**

| Bouillon cube factory startup costs | 2021 | 2022 |
| --- | --- | --- |
| *Equipment* |  |  |
| Feeder (unit cost) and installation | $5,000 |  |
| Number of factories requiring a feeder | 3 |  |
| Total feeder cost | $15,000 |  |
| iCheck portable fluorometer | $8,350 |  |
| iCheck portable photometer | $2,950 |  |
| Manual centrifuge | $476 |  |
| Number of factories requiring a fluorometer | 3 |  |
| Number of factories requiring a photometer | 3 |  |
| Number of factories requiring a centrifuge | 3 |  |
| Total fluorometer, photometer, and centrifuge costs | $35,328 |  |
| Tax or duty on factory equipment (%) | 10% |  |
| Equipment shipping (%) | 10% |  |
| VAT (%) | 18% |  |
| Total equipment cost | $69,453 |  |
| *Labeling* |  |  |
| Redesign of labels and label printing plates (unit cost) | $327 |  |
| Number of factories/brands requiring label and printing plates redesign | 3 |  |
| Total labeling cost | $981 |  |
| *Training* |  |  |
| Training and sensitization for fortification and quality assurance personnel (unit cost per training session) | $249 | $249 |
| Number of training sessions per factory | 3 | 3 |
| Total training cost | $2,242 | $2,242 |
| TOTAL factory start-up costs | $72,676 | $2,242 |
| Average start-up costs per factory | $24,225 | $747 |

Source: Authors’ calculations. All monetary values are expressed in 2021 USD.

**Supplementary Table 7: Operational Costs for Factories, Senegal**

| Annual factory operating costs | 2023 | 2024 | 2025 | 2026 |
| --- | --- | --- | --- | --- |
| *Fortification* |  |  |  |  |
| FTE additional labor for production/premix-related activities | 1 | 1 | 1 | 1 |
| Total labor cost for production/premix-related activities | $11,934 | $11,934 | $11,934 | $11,934 |
| Fortification equipment maintenance (% of equipment cost) | 7% | 7% | 7% | 7% |
| Total equipment maintenance cost | $2,633 | $2,633 | $2,633 | $2,633 |
| Total fortification cost | $14,567 | $14,567 | $14,567 | $14,567 |
| *Internal quality assurance/quality control (QA/QC)* |  |  |  |  |
| Portable photometer supplies - reagents for in-house chemical analysis (unit cost per test) | $6 | $6 | $6 | $6 |
| Number of in-house tests for iron per day per factory | 0 | 0 | 0 | 0 |
| Portable fluorometer supplies - reagents for in-house chemical analysis (unit cost per test) | $8 | $8 | $8 | $8 |
| Number of in-house tests for vitamin A per day per factory | 3 | 3 | 3 | 3 |
| Total reagents cost | $18,000 | $18,000 | $18,000 | $18,000 |
| FTE per factory for personnel for QA/QC activities | 1 | 1 | 1 | 1 |
| Total QA/QC personnel cost | $14,917 | $14,917 | $14,917 | $14,917 |
| Total quality assurance/quality control cost | $32,917 | $32,917 | $32,917 | $32,917 |
| *External quality assurance/quality control (QA/QC)* |  |  |  |  |
| External lab quantitative testing (unit cost per sample tested including administration, packaging, transport/shipping, etc.) | $62 | $62 | $62 | $62 |
| Number of external lab tests per factory per year | 36 | 36 | 36 | 36 |
| Total quality assurance/quality control cost | $2,242 | $2,242 | $2,242 | $2,242 |
| *Training/Retraining* |  |  |  |  |
| Training/retraining for fortification and quality assurance personnel (unit cost per training session) | $249 | $249 | $249 | $249 |
| Number of training/retraining sessions per factory per year | 1 | 1 | 1 | 1 |
| Total training/retraining cost | $747 | $747 | $747 | $747 |
| *Management, overhead, administration* |  |  |  |  |
| Management, overhead, administration (as % of premix cost) | 5% | 5% | 5% | 5% |
| Total management, overhead, administration cost | $16,658 | $17,090 | $17,528 | $17,972 |
| Total annual factory operating costs | $67,132 | $67,563 | $68,002 | $68,446 |
| Average annual operating costs per factory | $22,377 | $22,521 | $22,667 | $22,815 |

Source: Authors’ calculations. All monetary values are expressed in 2021 USD.

**Supplementary Table 8: Operational Costs for Factories, Burkina Faso**

| Annual factory operating costs | 2023 | 2024 | 2025 | 2026 |
| --- | --- | --- | --- | --- |
| *Fortification* |  |  |  |  |
| FTE additional labor for production/premix-related activities | 1 | 1 | 1 | 1 |
| Total labor cost for production/premix-related activities | $7,729 | $7,822 | $7,916 | $8,011 |
| Fortification equipment maintenance (% of equipment cost) | 7% | 7% | 7% | 7% |
| Total equipment maintenance cost | $3,523 | $3,523 | $3,523 | $3,523 |
| Total fortification cost | $11,252 | $11,345 | $11,439 | $11,534 |
| *Internal quality assurance/quality control (QA/QC)* |  |  |  |  |
| Portable photometer supplies - reagents for in-house chemical analysis (unit cost per test) | $6 | $6 | $6 | $6 |
| Number of in-house tests for iron per day per factory | 0 | 0 | 0 | 0 |
| Portable fluorometer supplies - reagents for in-house chemical analysis (unit cost per test) | $7 | $7 | $7 | $7 |
| Number of in-house tests for vitamin A per day per factory | 3 | 3 | 3 | 3 |
| Total reagents cost | $15,975 | $15,975 | $15,975 | $15,975 |
| FTE per factory for personnel for QA/QC activities | 1 | 1 | 1 | 1 |
| Total QA/QC personnel cost | $9,433 | $9,433 | $9,433 | $9,433 |
| Total quality assurance/quality control cost | $25,408 | $25,408 | $25,408 | $25,408 |
| *External quality assurance/quality control (QA/QC)* |  |  |  |  |
| External lab quantitative testing (unit cost per sample tested including administration, packaging, transport/shipping, etc.) | $62 | $62 | $62 | $62 |
| Number of external lab tests per factory per year | 36 | 36 | 36 | 36 |
| Total quality assurance/quality control cost | $2,242 | $2,242 | $2,242 | $2,242 |
| *Training/Retraining* |  |  |  |  |
| Training/retraining for fortification and quality assurance personnel (unit cost per training session) | $249 | $249 | $249 | $249 |
| Number of training/retraining sessions per factory per year | 1 | 1 | 1 | 1 |
| Total training/retraining cost | $747 | $747 | $747 | $747 |
| *Management, overhead, administration* |  |  |  |  |
| Management, overhead, administration (as % of premix cost) | 5% | 5% | 5% | 5% |
| Total management, overhead, administration cost | $12,241 | $12,581 | $12,928 | $13,281 |
| Total annual factory operating costs | $51,891 | $52,323 | $52,764 | $53,213 |
| Average annual operating costs per factory | $17,297 | $17,441 | $17,588 | $17,738 |

Source: Authors’ calculations. All monetary values are expressed in 2021 USD.

**Supplementary Table 9: Start-up Costs for Government; Senegal**

| Government bouillon cube startup costs | 2021 | 2022 |
| --- | --- | --- |
| *Planning* |  |  |
| Baseline survey | $249,131 | $50,000 |
| Industrial assessment | $15,000 | $0 |
| Formulation of standards/norms | $20,000 | $20,000 |
| Development of monitoring and evaluation (M&E) plan | $10,000 | $10,000 |
| Total planning cost | $294,131 | $80,000 |
| *Equipment* |  |  |
| iCheck portable fluorometer (vitamin A) | $8,690 |  |
| iCheck portable photometer (Iron) | $3,373 |  |
| Manual centrifuge | $476 |  |
| Total number of fluorometers | 4 |  |
| Total number of photometers | 0 |  |
| Total number of manual centrifuges | 4 |  |
| Total iCheck and manual centrifuge costs | $36,664 |  |
| Tax or duty on equipment (%) | 10% |  |
| Equipment shipping (%) | 10% |  |
| VAT (%) | 15% |  |
| Total equipment cost | $49,496 | $0 |
| *Social marketing/advocacy* |  |  |
| Social marketing/advocacy campaign costs | $50,000 | $50,000 |
| Total social marketing/advocacy cost | $50,000 | $50,000 |
| *Training* |  |  |
| Cost of training sessions for food control agency/program monitors, lab technicians, supervisors (unit cost) | $4,983 | $4,983 |
| Number of training sessions | 5 | 5 |
| Total training cost | $24,913 | $24,913 |
| TOTAL government start-up costs | $418,540 | $154,913 |

Source: Authors’ calculations. All monetary values are expressed in 2021 USD.

**Supplementary Table 10: Annual Operational Costs for Government, Part 1, Senegal**

| Annual government recurring costs | 2023 | 2024 | 2025 | 2026 |
| --- | --- | --- | --- | --- |
| *Factory inspections and monitoring* |  |  |  |  |
| Cost per factory inspection (unit cost - includes labor, travel, incidentals, management, etc.) | $162 | $162 | $162 | $162 |
| Number of inspections per year | 36 | 36 | 36 | 18 |
| Total cost of inspections | $5,830 | $5,830 | $5,830 | $2,915 |
| Portable fluorometer supplies - reagents for sample analysis (unit cost per test) | $8 | $8 | $8 | $8 |
| Number of samples analyzed for vitamin A per factory inspection | 5 | 5 | 5 | 5 |
| Total cost of supplies for vitamin A analyses | $1,440 | $1,440 | $1,440 | $720 |
| Portable photometer supplies - reagents for sample analysis (unit cost per test) | $6 | $6 | $6 | $6 |
| Number of samples analyzed for iron per factory inspection | 0 | 0 | 0 | 0 |
| Total cost of supplies for iron analyses | $0 | $0 | $0 | $0 |
| Total cost of analysis of factory samples | $1,440 | $1,440 | $1,440 | $720 |
| Total factory inspections and monitoring cost | $7,270 | $7,270 | $7,270 | $3,635 |
| *Import monitoring* |  |  |  |  |
| FTE for import monitoring activities per port of entry | 0.50 | 0.50 | 0.50 | 0.50 |
| Total labor cost of import monitoring | $4,972 | $4,972 | $4,972 | $4,972 |
| Portable fluorometer supplies - reagents for sample analysis (unit cost per test) | $8 | $8 | $8 | $8 |
| Number of samples analyzed for vitamin A per consignment/delivery inspection | 2 | 2 | 2 | 2 |
| Total cost of supplies for vitamin A analyses | $763 | $763 | $763 | $763 |
| Portable photometer supplies - reagents for sample analysis (unit cost per test) | $6 | $6 | $6 | $6 |
| Number of samples analyzed for iron per consignment/delivery inspection | 0 | 0 | 0 | 0 |
| Total cost of supplies for iron analyses |  |  |  |  |
| Total cost of analysis of import samples | $763 | $763 | $763 | $763 |
| Total import monitoring cost | $5,735 | $5,735 | $5,735 | $5,735 |

Source: Authors’ calculations. All monetary values are expressed in 2021 USD.

**Supplementary Table 11: Annual Operational Costs for Government, Part 1, Burkina Faso**

| Annual government recurring costs | 2023 | 2024 | 2025 | 2026 |
| --- | --- | --- | --- | --- |
| *Factory inspections and monitoring* |  |  |  |  |
| Cost per factory inspection (unit cost - includes labor, travel, incidentals, management, etc.) | $162 | $162 | $162 | $162 |
| Number of inspections per year | 36 | 36 | 36 | 18 |
| Total cost of inspections | $5,830 | $5,830 | $5,830 | $2,915 |
| Portable fluorometer supplies - reagents for sample analysis (unit cost per test) | $7 | $7 | $7 | $7 |
| Number of samples analyzed for vitamin A per factory inspection | 5 | 5 | 5 | 5 |
| Total cost of supplies for vitamin A analyses | $1,278 | $1,278 | $1,278 | $639 |
| Portable photometer supplies - reagents for sample analysis (unit cost per test) | $6 | $6 | $6 | $6 |
| Number of samples analyzed for iron per factory inspection | 0 | 0 | 0 | 0 |
| Total cost of supplies for iron analyses | $0 | $0 | $0 | $0 |
| Total cost of analysis of factory samples | $1,278 | $1,278 | $1,278 | $639 |
| Total factory inspections and monitoring cost | $7,108 | $7,108 | $7,108 | $3,554 |
| *Import monitoring* |  |  |  |  |
| FTE for import monitoring activities per port of entry | 0.50 | 0.50 | 0.50 | 0.50 |
| Total labor cost of import monitoring | $3,220 | $3,259 | $3,298 | $3,338 |
| Portable fluorometer supplies - reagents for sample analysis (unit cost per test) | $7 | $7 | $7 | $7 |
| Number of samples analyzed for vitamin A per consignment/delivery inspection | 2 | 2 | 2 | 2 |
| Total cost of supplies for vitamin A analyses | $710 | $710 | $710 | $710 |
| Portable photometer supplies - reagents for sample analysis (unit cost per test) | $6 | $6 | $6 | $6 |
| Number of samples analyzed for iron per consignment/delivery inspection | 0 | 0 | 0 | 0 |
| Total cost of supplies for iron analyses |  |  |  |  |
| Total cost of analysis of import samples | $710 | $710 | $710 | $710 |
| Total import monitoring cost | $3,930 | $3,969 | $4,008 | $4,048 |

Source: Authors’ calculations. All monetary values are expressed in 2021 USD.

**Supplementary Table 12: Annual Operational Costs for Government, Part 2, Senegal**

| Annual government recurring costs | 2023 | 2024 | 2025 | 2026 |
| --- | --- | --- | --- | --- |
| *Commercial monitoring/Market Surveys* |  |  |  |  |
| FTE for commercial monitoring at markets/retail outlets | 1 | 1 | 1 | 1 |
| Total cost of labor for commercial monitoring | $4,972.42 | $4,972.42 | $4,972.42 | $4,972.42 |
| Portable fluorometer supplies - reagents for sample analysis (unit cost per test) | $8 | $8 | $8 | $8 |
| Number of samples analyzed for vitamin A per commercial outlet visit | 2 | 2 | 2 | 2 |
| Total cost of supplies for vitamin A analyses | $4,578 | $4,578 | $4,578 | $4,578 |
| Portable photometer supplies - reagents for sample analysis (unit cost per test) | $6 | $6 | $6 | $6 |
| Number of samples analyzed for iron per commercial visit | 0 | 0 | 0 | 0 |
| Total cost of supplies for iron analyses | $0 | $0 | $0 | $0 |
| Total cost of chemical analysis of commercial samples | $4,578 | $4,578 | $4,578 | $4,578 |
| Total commercial monitoring cost | $9,550 | $9,550 | $9,550 | $9,550 |
| *Household monitoring* |  |  |  |  |
| Cost of new data collection or adding questions to existing annual surveys | $20,000 | $20,000 | $20,000 | $20,000 |
| Cost of data analysis | $10,000 | $10,000 | $10,000 | $10,000 |
| Total household monitoring cost | $0 | $30,000 | $0 | $0 |
| *Social marketing* |  |  |  |  |
| Annual cost of social marketing, education, communication | $10,000 | $10,000 | $10,000 | $10,000 |
| Total social marketing cost | $10,000 | $10,000 | $10,000 | $10,000 |
| *Nutrition Surveillance (impact evaluation)* |  |  |  |  |
| Cost of national nutrition survey - data collection | $500,000 | $500,000 | $500,000 | $500,000 |
| Cost of national nutrition survey - data analysis | $50,000 | $50,000 | $50,000 | $50,000 |
| Total nutrition surveillance cost | $0 | $0 | $0 | $0 |
| *Training/Retraining* |  |  |  |  |
| Cost of training/retraining sessions for food control agency/program monitors, lab technicians, supervisors (unit cost) | $4,983 | $4,983 | $4,983 | $4,983 |
| Number of training/retraining sessions per year per factory | 1 | 1 | 1 | 1 |
| Total training/retraining cost | $14,948 | $14,948 | $14,948 | $14,948 |
| *Management, overhead, administration* |  |  |  |  |
| Management, overhead, administration (% of monitoring cost) | 5% | 5% | 5% | 5% |
| Total maintenance cost | $1,128 | $2,628 | $1,128 | $946 |
| Total annual government operating costs | $48,631 | $80,131 | $48,631 | $44,815 |

Source: Authors’ calculations. All monetary values are expressed in 2021 USD.

**Supplementary Table 13: Annual Operational Costs for Government, Part 2, Burkina Faso**

| Annual government recurring costs | 2023 | 2024 | 2025 | 2026 |
| --- | --- | --- | --- | --- |
| *Commercial monitoring/Market Surveys* |  |  |  |  |
| FTE for commercial monitoring at markets/retail outlets | 1 | 1 | 1 | 1 |
| Total cost of labor for commercial monitoring | $3,220 | $3,259 | $3,298 | $3,338 |
| Portable fluorometer supplies - reagents for sample analysis (unit cost per test) | $8 | $8 | $8 | $8 |
| Number of samples analyzed for vitamin A per commercial outlet visit | 2 | 2 | 2 | 2 |
| Total cost of supplies for vitamin A analyses | $4,578 | $4,578 | $4,578 | $4,578 |
| Portable photometer supplies - reagents for sample analysis (unit cost per test) | $6 | $6 | $6 | $6 |
| Number of samples analyzed for iron per commercial visit | 0 | 0 | 0 | 0 |
| Total cost of supplies for iron analyses | $0 | $0 | $0 | $0 |
| Total cost of chemical analysis of commercial samples | $4,578 | $4,578 | $4,578 | $4,578 |
| Total commercial monitoring cost | $7,798 | $7,837 | $7,876 | $7,916 |
| *Household monitoring* |  |  |  |  |
| Cost of new data collection or adding questions to existing annual surveys | $20,000 | $20,000 | $20,000 | $20,000 |
| Cost of data analysis | $10,000 | $10,000 | $10,000 | $10,000 |
| Total household monitoring cost | $0 | $30,000 | $0 | $0 |
| *Social marketing* |  |  |  |  |
| Annual cost of social marketing, education, communication | $10,000 | $10,000 | $10,000 | $10,000 |
| Total social marketing cost | $10,000 | $10,000 | $10,000 | $10,000 |
| *Nutrition Surveillance (impact evaluation)* |  |  |  |  |
| Cost of national nutrition survey - data collection | $500,000 | $500,000 | $500,000 | $500,000 |
| Cost of national nutrition survey - data analysis | $50,000 | $50,000 | $50,000 | $50,000 |
| Total nutrition surveillance cost | $0 | $0 | $0 | $0 |
| *Training/Retraining* |  |  |  |  |
| Cost of training/retraining sessions for food control agency/program monitors, lab technicians, supervisors (unit cost) | $4,983 | $4,983 | $4,983 | $4,983 |
| Number of training/retraining sessions per year per factory | 1 | 1 | 1 | 1 |
| Total training/retraining cost | $14,948 | $14,948 | $14,948 | $14,948 |
| *Management, overhead, administration* |  |  |  |  |
| Management, overhead, administration (% of monitoring cost) | 5% | 5% | 5% | 5% |
| Total maintenance cost | $942 | $2,446 | $950 | $776 |
| Total annual government operating costs | $44,726 | $76,307 | $44,889 | $41,241 |

Source: Authors’ calculations. All monetary values are expressed in 2021 USD.

**Supplementary Table 14: Results of Cost Sensitivity Analyses**

| **Summary Cost Measures** | Sensitivity Analyses Scenarios | | | |
| --- | --- | --- | --- | --- |
|  | *Preferred Estimates* | *Fortificant Price Uncertainty*  *(all fortificant prices up/down 20%)* | *Premix VAT Uncertainty*  *(VAT eliminated)* | *Uncertainty around Bouillon Consumption Patterns*  *(Average bouillon consumption up/down 20%)* |
| **Nigeria** |  |  |  |  |
| Total Costs |  |  |  |  |
| Total 10-year Costs | $642,733,105 | $760,327,308  $525,138,902 | $545,934,589 | $770,443,738  $515,022,472 |
| Start-up Costs (2 years) | $910,730 | $910,730  $910,730 | $910,730 | $910,730  $910,370 |
| Non-premix Operational Costs (8 years) | $31,207,812 | $36,353,749  $26,061,874 | $26,598,359 | $36,795,532  $25,620,092 |
| Premix Costs (8 years) | $610,614,563 | $723,062,829  $498,166,297 | $518,425,500 | $732,737,476  $488,491,650 |
| Unit Costs |  |  |  |  |
| Cost per MT of Fortified Bouillon | $333 | $394  $272 | $283 | $333  $334 |
| Cost per Consumer Reached | $0.34 | $0.40  $0.28 | $0.29 | $0.40  $0.27 |
| **Burkina Faso** |  |  |  |  |
| Total Costs |  |  |  |  |
| Total 10-year Costs | $20,770,738 | $24,335,233  $17,206,243 | $20,322,240 | $24,644,199  $16,897,277 |
| Start-up Costs (2 years) | $647,595 | $647,595  $647,595 | $647,595 | $647,595  $647,595 |
| Non-premix Operational Costs (8 years) | $863,669 | $883,538  $843,800 | $842,311 | $885,235  $842,102 |
| Premix Costs (8 years) | $19,259,475 | $22,804,101  $15,714,849 | $18,832,334 | $23,111,370  $15,407,580 |
| Unit Costs |  |  |  |  |
| Cost per MT of Fortified Bouillon | $323 | $378  $268 | $316 | $319  $328 |
| Cost per Consumer Reached | $0.13 | $0.15  $0.10 | $0.12 | $0.15  $0.10 |
| **Senegal** |  |  |  |  |
| Total Costs |  |  |  |  |
| Total 10-year Costs | $27,689,836 | $32,506,646  $22,873,026 | $27,083,767 | $32,924,162  $22,455,511 |
| Start-up Costs (2 years) | $649,952 | $649,952  $649,952 | $649,952 | $649,952  $649,952 |
| Non-premix Operational Costs (8 years) | $1,013,971 | $1,040,820  $987,121 | $985,110 | $1,043,114  $984,828 |
| Premix Costs (8 years) | $26,025,914 | $30,815,874  $21,235,953 | $25,448,705 | $31,231,097  $20,820,731 |
| Unit Costs |  |  |  |  |
| Cost per MT of Fortified Bouillon | $319 | $374  $263 | $312 | $316  $323 |
| Cost per Consumer Reached | $0.20 | $0.23  $0.16 | $0.19 | $0.23  $0.16 |

Source: Authors’ calculations. All monetary values are expressed in 2021 USD.

**Supplementary Figure 1: Government Start-up Costs of Bouillon Fortification, by Cost Category, Senegal, 30% of Codex NRVs**


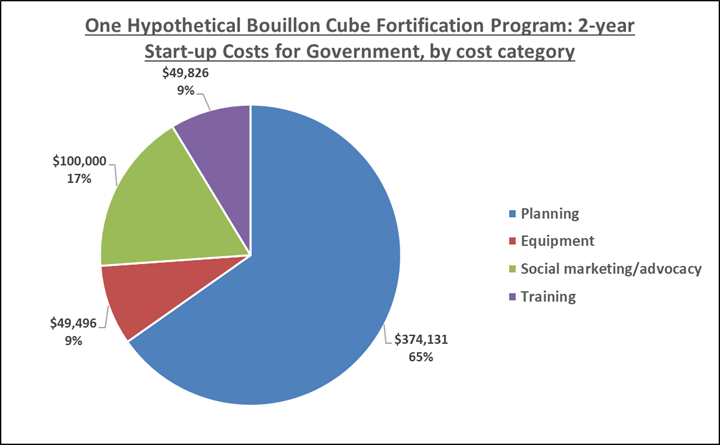


Source: Authors’ calculations. All monetary values are expressed in 2021 USD.

**Supplementary Figure 2: Industry Start-up Costs of Bouillon Fortification, by Cost Category, Senegal, 30% of Codex NRVs**


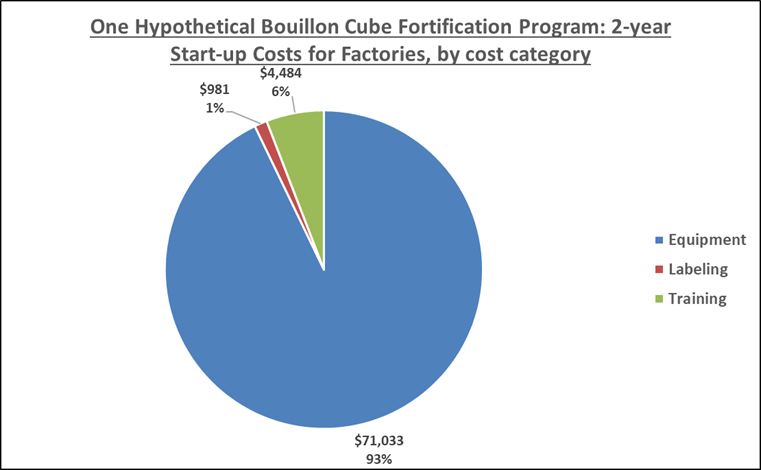


Source: Authors’ calculations. All monetary values are expressed in 2021 USD.

**Supplementary Figure 3: Government Non-premix Operational Costs of Bouillon Fortification, by Cost Category, Senegal, 30% of Codex NRVs**


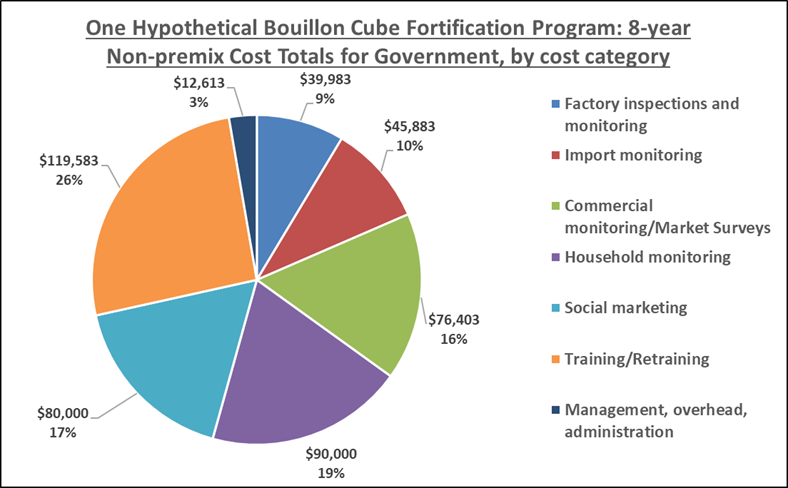


Source: Authors’ calculations. All monetary values are expressed in 2021 USD.

**Supplementary Figure 4: Industry Non-premix Operational Costs of Bouillon Fortification, by Cost Category, Senegal, 30% of Codex NRVs**


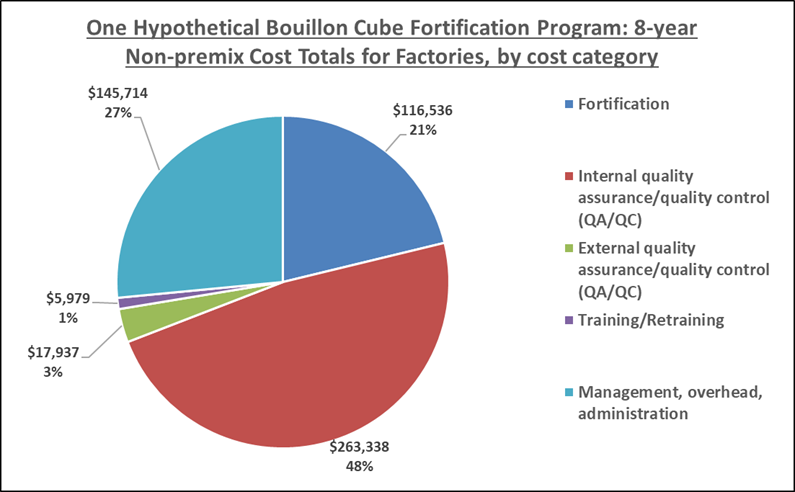


Source: Authors’ calculations. All monetary values are expressed in 2021 USD.

**Supplementary Figure 5: Operational-Year Costs of Bouillon Fortification, by Cost Category, 2026, Senegal, 30% of Codex NRVs**


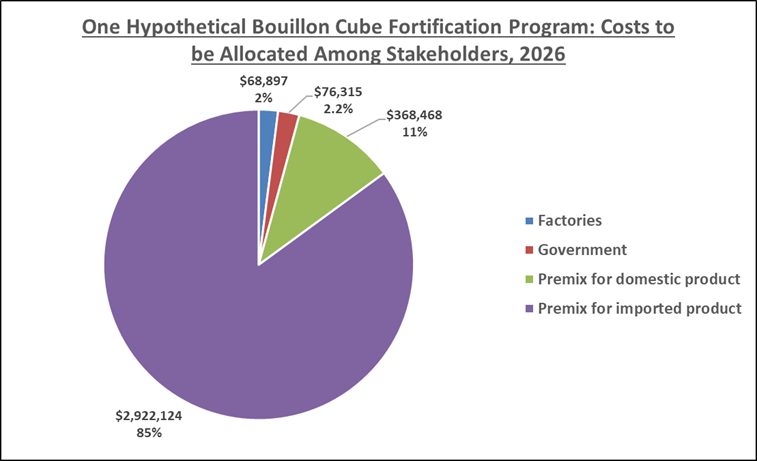


Source: Authors’ calculations. All monetary values are expressed in 2021 USD.

**Supplementary Figure 6: Government Start-up Costs of Bouillon Fortification, by Cost Category, Burkina Faso, 30% of Codex NRVs**


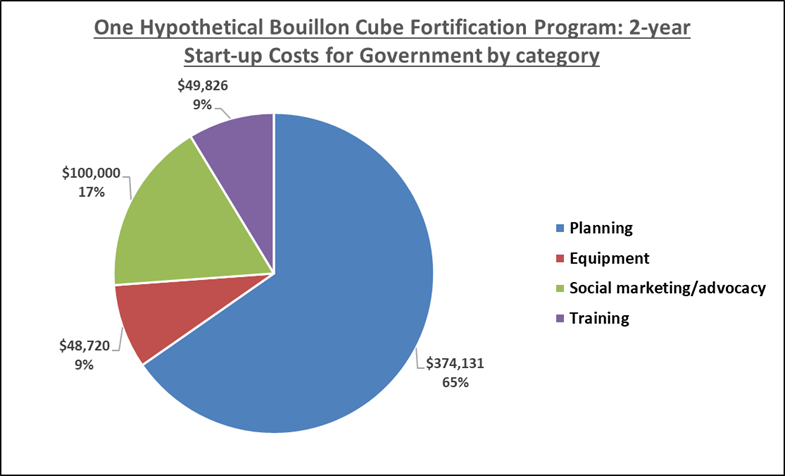


Source: Authors’ calculations. All monetary values are expressed in 2021 USD.

**Supplementary Figure 7: Industry Start-up Costs of Bouillon Fortification, by Cost Category, Burkina Faso, 30% of Codex NRVs**


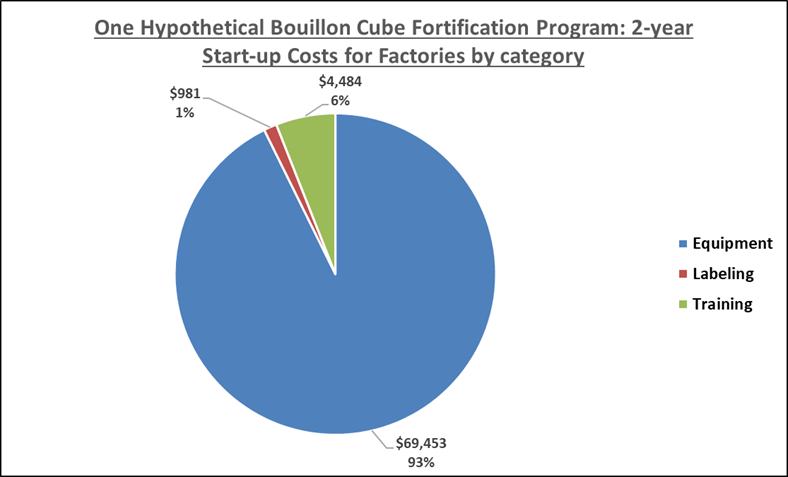


Source: Authors’ calculations. All monetary values are expressed in 2021 USD.

**Supplementary Figure 8: Government Non-premix Operational Costs of Bouillon Fortification, by Cost Category, Burkina Faso, 30% of Codex NRVs**


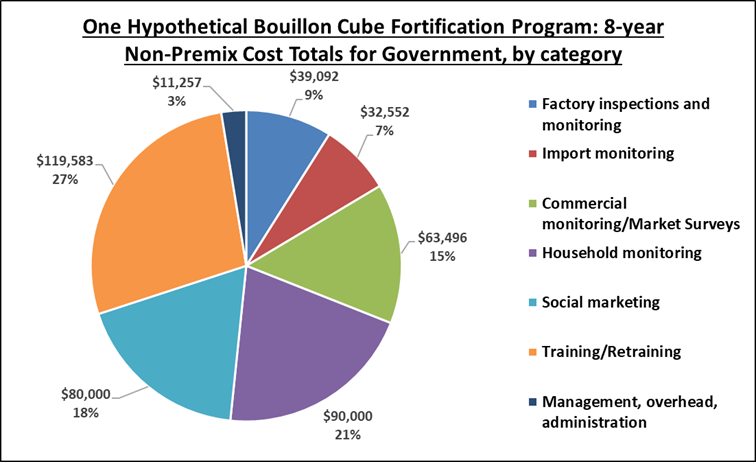


Source: Authors’ calculations. All monetary values are expressed in 2021 USD.

**Supplementary Figure 9: Industry Non-premix Operational Costs of Bouillon Fortification, by Cost Category, Burkina Faso, 30% of Codex NRVs**


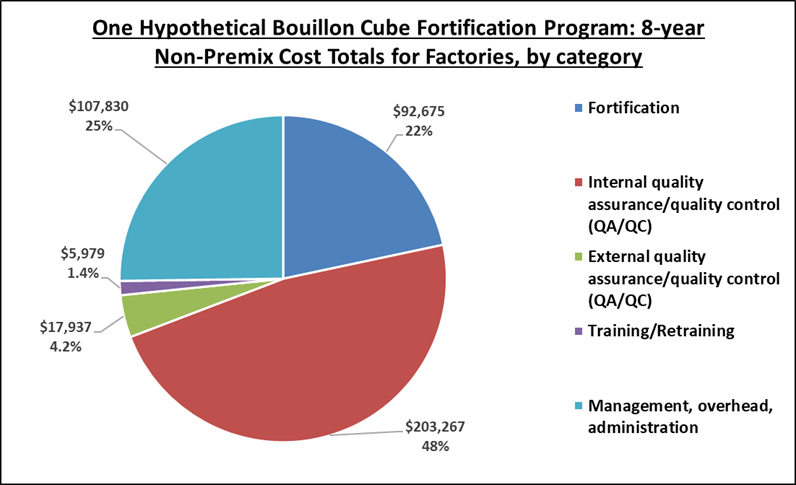


Source: Authors’ calculations. All monetary values are expressed in 2021 USD.

**Supplementary Figure 10: Operational-Year Costs of Bouillon Fortification, by Cost Category, 2026, Burkina Faso, 30% of Codex NRVs**


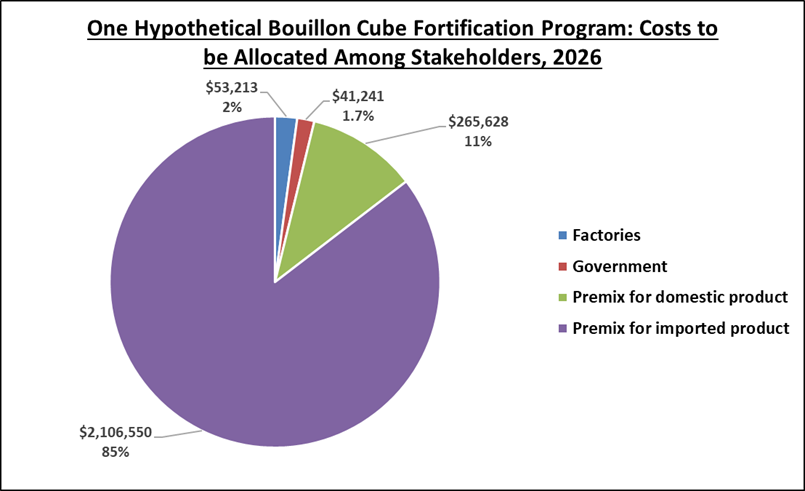


Source: Authors’ calculations. All monetary values are expressed in 2021 USD.
